# Author Correction: Short- and long-range interactions in the HIV-1 5′ UTR regulate genome dimerization and packaging

**DOI:** 10.1038/s41594-023-00946-4

**Published:** 2023-02-24

**Authors:** Liqing Ye, Anne-Sophie Gribling-Burrer, Patrick Bohn, Anuja Kibe, Charlene Börtlein, Uddhav B. Ambi, Shazeb Ahmad, Marco Olguin-Nava, Maureen Smith, Neva Caliskan, Max von Kleist, Redmond P. Smyth

**Affiliations:** 1 Helmholtz Institute for RNA-based Infection Research, Helmholtz Centre for Infection Research, Würzburg, Germany; 2 Faculty of Medicine, University of Würzburg, Würzburg, Germany; 3 P5 Systems Medicine of Infectious Disease, Robert Koch-Institute, Berlin, Germany

**Keywords:** Virology, Structure determination, Riboswitches, RNA

Correction to: *Nature Structural & Molecular Biology* 10.1038/s41594-022-00746-2. Published online 28 March 2022.

In the version of this article initially published, in the ‘Library preparation’ subsection of Methods, the sentence beginning “For each binding experiment…” omitted 0.2 mg ml^−1^ of yeast tRNA from the recipe for high salt buffer. The error has been corrected in the HTML and PDF versions of the article.

