# Supplementary material for: Short- and long-range interactions in the HIV-1 5′ UTR regulate genome dimerization and packaging
Source: Nat Struct Mol Biol. 2022 Mar 28;29(4):306–19. doi: 10.1038/s41594-022-00746-2 (PMC9010304; doi:10.1038/s41594-022-00746-2)
Supplement: Supplementary file 1 — Mathematical and statistical methods for analyzing mutational interference data in dimerization experiments. [file 41594_2022_746_MOESM1_ESM.pdf]

nature portfolio

https://doi.org/10.1038/s41594-022-00746-2

Supplementary information

Short- and long-range interactions in the HIV-1 5′ UTR regulate genome dimerization and packaging

In the format provided by the authors and unedited

Mathematical & statistical methods for analysing mutational interference data in dimerisation experiments

In this note, we describe the methods to infer the functional contribution of mutations with regards to dimerisation, extending on previous ideas [1,2]. Specifically, the input for analysis consists of base counts at each position on the monomer-, as well as the dimer samples of RNAs (see Fig. 1). The mathematical methods then allow (i) translating these counts into a quantitative effect associated with each mutation m at each nucleotide position i. This quantitative output is a change in dimerisation affinity of a particular mutation m at nucleotide position i, relative to the wild type w, denoted by $K_m^{\mathrm{dimer}}(i) = \frac{\mathrm{Kd}_m}{\mathrm{Kd}_w}(i)$, where Kd denotes the overall dissociation constant between two molecules of the RNA. This measure will be evaluated for all possible mutations and all nucleotide positions. Obviously, the dimerisation domain in the RNA is the area where mutations have the strongest impact on dimerisation affinity. Secondly, (ii) the statistical significance of the $K^{\mathrm{dimer}}(i)$ estimates is assessed. To do this, we use a non-parametric re-sampling-like procedure. A sketch of the analysis procedure is shown in Figure 1.

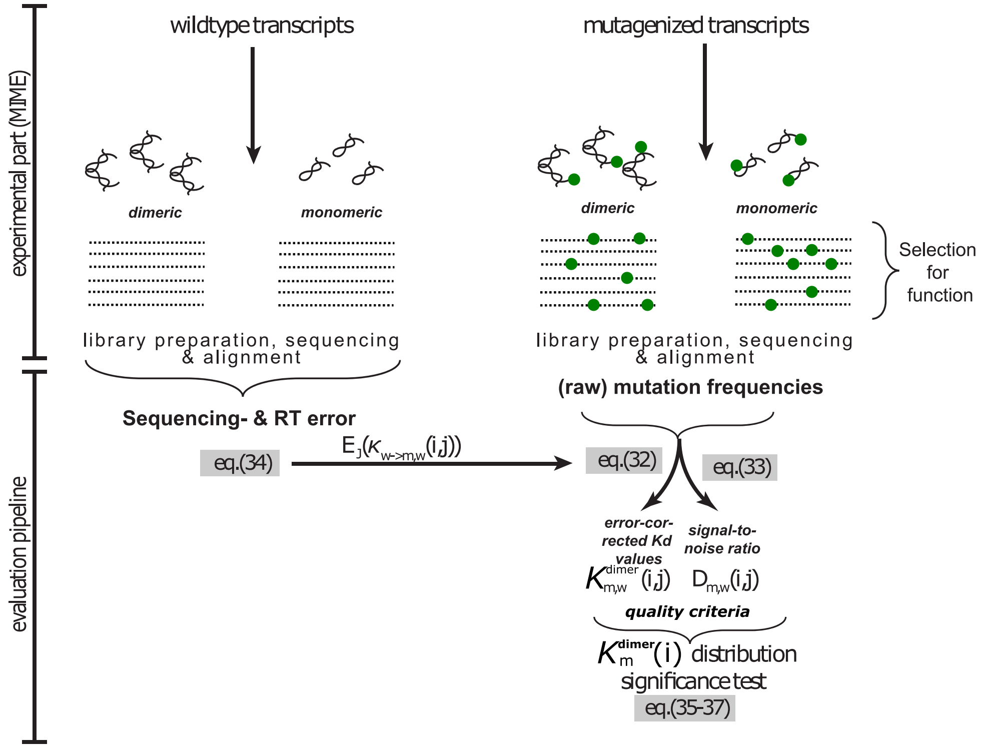

Figure 1. Mathematical analysis pipeline. Important quantities and equations are highlighted and can be found within this document. In brief: wild type libraries are sequenced after functional separation (dimer/monomer). From the dimer and monomer samples, position- and mutation specific detection errors $\mathbb{E}_J(\kappa_{w\to m,w}(i,j))$ (combined RT- & sequencing error) are derived. They are subsequently used to correct re-sampled relative dimerisation affinities K^{dimer} from the mutant libraries and to derive a signal-to-noise ratio for each position i, co-varying position j and mutation m. The signal-to-noise ratio, together with additional quality criteria can be used to detect and filter out unreliable/insufficient signals. The analysis pipeline yields the re-sampling distribution for $K_m^{\mathrm{dimer}}(i)$ and its statistical ascertainment.

This document is organized as follows:

1. We derive the mathematical concepts that translate the sequencing output into relative dimerisation affinities K^{dimer}. Next, we quantify sequencing and reverse transcription errors. The K^{dimer} values are then corrected for these errors and for each mutation m and nucleotide position i, a signal-to-noise ratio $D_m(i)$ is derived. Note, that this procedure yields one point estimate for each mutation m at each nucleotide position i.

2. In order to assess the statistical certainty of the K^{dimer} estimates, a re-sampling procedure is employed. The utilised resampling procedure is based on the analysis of pairs of positions, but is otherwise based on the methods stated above. In the main manuscript, we report the results from the re-sampling analysis. The analysis procedure referring is shown in Figure 1.

3. We discuss the theoretical possibility of dimerisation of two compensatory mutants, which, in theory, could affect the estimation of the quantitative binding affinity. However, we show that this possibility is vanishingly small and there is no need to take these cases into account.

1 Mathematical & statistical concepts

1.1 Relation between nucleotide frequency and relative dimerisation affinity

The basic reaction scheme underlying the competitive binding experiment that separates RNA by dimerization affinity is shown in Fig. 2 (left). In the graphic, the differentially colored ★ symbols indicate the presence of a particular mutation at a specific nucleotide position in the RNA.

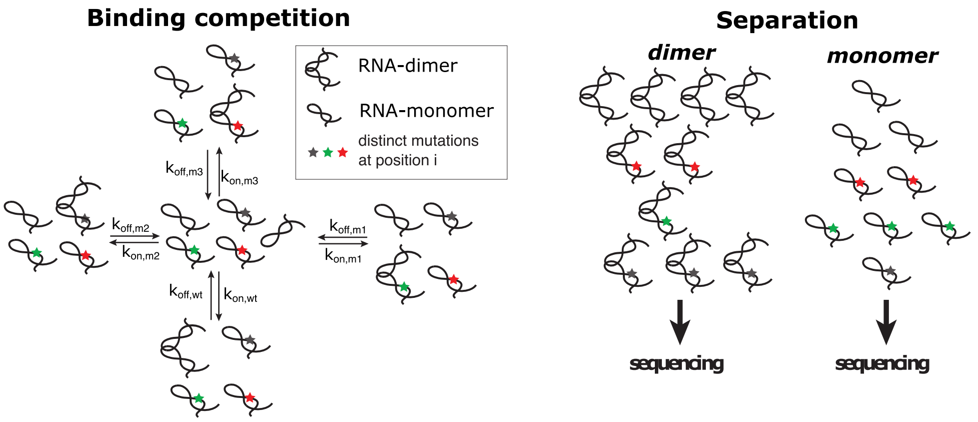

Figure 2. Reaction scheme underlying the competition experiment that selects RNA by dimerization affinity (left). Dimers and monomers are separated, prepared for sequencing and sequenced to obtain mutation frequencies (right) at each position.

If we assumed that *wild type* RNA is way more abundant than mutated RNA, and hence $\mathcal{S}_w(i, \text{monomer}) \approx \sum_{n\in\{m_1,m_2,m_3,w\}} \mathcal{S}_n(i, \text{monomer})$, the mass-action kinetics describing this experiment are given by:

$$
\begin{aligned}
\frac{\mathrm{d}}{\mathrm{d}t}\mathcal{S}_w(\mathrm{i}, \text{dimer}) &= \big(\mathcal{S}_w(i, \text{monomer})\big)^2 \cdot k_{\text{on},w}(i) - \mathcal{S}_w(i, \text{dimer}) \cdot k_{\text{off},w}(i) && (1)\\
\frac{\mathrm{d}}{\mathrm{d}t}\mathcal{S}_w(i, \text{monomer}) &= -\big(\mathcal{S}_w(i, \text{monomer})\big)^2 \cdot k_{\text{on},w}(i) + \mathcal{S}_w(i, \text{dimer}) \cdot k_{\text{off},w}(i) && (2)\\
\frac{\mathrm{d}}{\mathrm{d}t}\mathcal{S}_{m1}(i, \text{dimer}) &= \mathcal{S}_{m1}(i, \text{monomer}) \cdot \mathcal{S}_w(i, \text{monomer}) \cdot k_{\text{on},m1}(i) - \mathcal{S}_{m1}(i, \text{dimer}) \cdot k_{\text{off},m1}(i) && (3)\\
\frac{\mathrm{d}}{\mathrm{d}t}\mathcal{S}_{m1}(i, \text{monomer}) &= -\mathcal{S}_{m1}(i, \text{monomer}) \cdot \mathcal{S}_w(i, \text{monomer}) \cdot k_{\text{on},m1}(i) + \mathcal{S}_{m1}(i, \text{dimer}) \cdot k_{\text{off},m1}(i) && (4)\\
\vdots &= \vdots \\
\frac{\mathrm{d}}{\mathrm{d}t}\mathcal{S}_{m3}(i, \text{monomer}) &= -\mathcal{S}_{m3}(i, \text{monomer}) \cdot \mathcal{S}_w(i, \text{monomer}) \cdot k_{\text{on},m3}(i) + \mathcal{S}_{m3}(i, \text{dimer}) \cdot k_{\text{off},m3}(i) && (5)
\end{aligned}
$$

where $\mathcal{S}_w(i, \text{dimer})$ denotes the concentration of dimerized RNA carrying a wild type base at nucleotide position i and $\mathcal{S}_w(i, \text{monomer})$ denotes the concentration of monomeric wild type RNA. Correspondingly, the subscripts $m1, ..., m3$ indicate the presence of one of the possible three mutations at nucleotide position i. The parameters $k_{\text{off}}(i), k_{\text{on}}(i)$ denote the respective rates of dissociation and association.
In a steady state condition, i.e. if we allow sufficient time to obtain a binding equilibrium, the left hand

side (the rate of change) of the equations becomes zero.

$$
\begin{aligned}
0 &= \big(\mathcal{S}_w(i,\text{monomer})\big)^2 \cdot k_{\text{on},w}(i) - \mathcal{S}_w(i,\text{dimer}) \cdot k_{\text{off},w}(i) && (6)\\
0 &= -\big(\mathcal{S}_w(i,\text{monomer})\big)^2 \cdot k_{\text{on},w}(i) + \mathcal{S}_w(i,\text{dimer}) \cdot k_{\text{off},w}(i) && (7)\\
0 &= \mathcal{S}_{m1}(i,\text{monomer}) \cdot \mathcal{S}_w(i,\text{monomer}) \cdot k_{\text{on},m1}(i) - \mathcal{S}_{m1}(i,\text{dimer}) \cdot k_{\text{off},m1}(i) && (8)\\
0 &= -\mathcal{S}_{m1}(i,\text{monomer}) \cdot \mathcal{S}_w(i,\text{monomer}) \cdot k_{\text{on},m1}(i) + \mathcal{S}_{m1}(i,\text{dimer}) \cdot k_{\text{off},m1}(i) && (9)\\
\vdots &= \vdots
\end{aligned}
$$

If we solve eq. (6) for the binding partner $\mathcal{S}_w(i,\text{monomer})$, we obtain

$$
\mathcal{S}_w(i,\text{monomer}) = \frac{\mathcal{S}_w(i,\text{dimer}) \cdot k_{\text{off},w}(i)}{\mathcal{S}_w(i,\text{monomer}) \cdot k_{\text{on},w}(i)} = \frac{\mathcal{S}_w(i,\text{dimer})}{\mathcal{S}_w(i,\text{monomer})} \cdot \text{Kd}_w(i), \tag{10}
$$

which we can substitute back into eq. (8). Note, that we replace the subscript $m1$ with m in the following, because this equation is generic for all possible mutations (m denotes one possible mutation from the set of all possible mutations $\{m1, m2, m3\}$).

$$
\begin{aligned}
0 &= \mathcal{S}_m(i,\text{monomer}) \cdot \frac{\mathcal{S}_w(i,\text{dimer})}{\mathcal{S}_w(i,\text{monomer})} \cdot \text{Kd}_w(i) \cdot k_{\text{on},m}(i) - \mathcal{S}_m(i,\text{dimer}) \cdot k_{\text{off},m}(i)\\
\mathcal{S}_m(i,\text{dimer}) \cdot k_{\text{off},m}(i) &= \mathcal{S}_m(i,\text{monomer}) \cdot \frac{\mathcal{S}_w(i,\text{dimer})}{\mathcal{S}_w(i,\text{monomer})} \cdot \text{Kd}_w(i) \cdot k_{\text{on},m}(i)\\
\frac{k_{\text{off},m}}{\text{Kd}_w \cdot k_{\text{on},m}}(i) &= \frac{\mathcal{S}_w(i,\text{dimer})}{\mathcal{S}_w(i,\text{monomer})} \cdot \frac{\mathcal{S}_m(i,\text{monomer})}{\mathcal{S}_m(i,\text{dimer})}
\end{aligned} \tag{11}
$$

$$
\boxed{\Rightarrow K_m^{\text{dimer}}(i) = \frac{\text{Kd}_m}{\text{Kd}_w}(i) = \frac{\mathcal{S}_w(i,\text{dimer})}{\mathcal{S}_w(i,\text{monomer})} \cdot \frac{\mathcal{S}_m(i,\text{monomer})}{\mathcal{S}_m(i,\text{dimer})}}, \tag{12}
$$

which denotes the impact of a particular mutation m (i.e. $A \to C$, $A \to G$ or $A \to U$ if the wild type base is adenosine) at position i in the RNA sequence on binding affinity.
Note: Unfortunately, the number of dimer/monomer sequences $\mathcal{S}$ are not known, instead we derive NGS reads $\mathcal{R}$, subject to errors $\mathcal{X}$ (sequencing and RT-errors). In order to account for this fact, we have to consider the relation between 'reads' and 'sequence numbers', which is exploited in error correction and justification of the proceeding steps.

1.2 Relation between 'Reads' and 'Sequence Numbers'

For any mutant nucleotide m at nucleotide position i, the number of NGS reads $\mathcal{R}_m(i)$ in the dimer/monomer samples is related with the RNA sequence numbers after protein capturing $\mathcal{S}_m(i)$ via

$$
\mathcal{R}_m(i) \quad = \quad \vartheta\Big(\mathcal{S}_m(i) - \sum_{n \neq m} \mathcal{X}_{m\to n}(i) + \sum_{n \neq m} \mathcal{X}_{n\to m}(i)\Big), \tag{13}
$$

where $\mathcal{X}_{m\to n}(i)$ is a random variable that denotes the number of sequences that are in fact nucleotide m, but were falsely detected as some other base n. Likewise, $\mathcal{X}_{n\to m}(i)$ is a random variable indicating the number of sequences that were originally some other nucleotide n, but were detected as m due to RT- and sequencing errors. Parameter ϑ denotes the normalization (relative titration) factor (if applied), which was used to normalise sample material of dimerised- and monomeric pools before next-generation

sequencing. For the ease of reading, we skipped the indicator 'dimer'/'monomer', since the equations apply in both cases. Consequently, we get

$$\mathcal{S}_m(i) \quad = \quad \frac{\mathcal{R}_m(i)}{\vartheta} + \sum_{n\neq m} \mathcal{X}_{m\to n}(i) - \sum_{n\neq m} \mathcal{X}_{n\to m}(i). \tag{14}$$

Since the wild type w is much more frequent in most samples (small per nucleotide mutation rate during RNA library preparation), i.e. $\mathcal{S}_w(i) \gg \mathcal{S}_m(i)$ and the error *probability* $\kappa_{n\to m}(i)$ (defined below) may not be vastly different for the distinct types of transitions $n \to m$, we have $\mathcal{X}_{m\to n}(i) \ll \mathcal{X}_{w\to m}(i) \gg \mathcal{X}_{k\to m}(i)$ for any $w \neq k \neq m$. Therefore, we can neglect all false detections, except those, where the wild type w was falsely detected as some mutant m. The expression simplifies accordingly:

$$\boxed{\mathcal{S}_m(i) \approx \frac{\mathcal{R}_m(i)}{\vartheta} - \mathcal{X}_{w\to m}(i)} \tag{15}$$

and likewise for the wild type

$$\mathcal{S}_w(i) \quad \approx \quad \frac{\mathcal{R}_w(i)}{\vartheta} + \sum_{m\in\mathbb{M}} \mathcal{X}_{w\to m}(i) \tag{16}$$

since $\kappa_{w\to m}(i) = \frac{\mathcal{X}_{w\to m}(i)}{\mathcal{S}_w(i)} \ll 1$ (the probability of false detection is small, typically $< 10^{-3}$ in Illumina machines) and $\vartheta \leq 1$ (only a fraction of the sample is taken for sequencing), the last equation simplifies further, as $\sum \mathcal{X}_{w\to m}(i) \ll \frac{\mathcal{R}_w(i)}{\vartheta}$:

$$\boxed{\mathcal{S}_w(i) \approx \frac{\mathcal{R}_w(i)}{\vartheta}.} \tag{17}$$

In relation to the inference of binding affinities, we can substitute the 'sequence numbers' with the NGS 'reads' (eqs. (15) and (17)) and obtain

$$K_m^{\text{dimer}}(i) \approx \frac{\mathcal{R}_w(i, \text{dimer})}{\mathcal{R}_w(i, \text{monomer})} \cdot \frac{\mathcal{R}_m(i, \text{monomer}) - \mathcal{X}_{w\to m}(i, \text{monomer}) \cdot \vartheta(\text{monomer})}{\mathcal{R}_m(i, \text{dimer}) - \mathcal{X}_{w\to m}(i, \text{dimer}) \cdot \vartheta(\text{dimer})}, \tag{18}$$

From the equation above, it is apparent that the relative dimerisation affinity $K_m^{\text{dimer}}(i)$ can only reliably be estimated when $\mathcal{R}_m(i) \gg \mathcal{X}_{w\to m}(i) \cdot \vartheta$, i.e. when the signal $\mathcal{R}_m$ is larger than the *noise* $\mathcal{X}$.

1.3 Error correction

In the following, we assume that the *noise* $\mathcal{X}$ is multinomially distributed, i.e.
$\mathcal{X}_{w\to m}(i) \sim \mathcal{M}\big(\mathcal{S}_w(i), \kappa_{w\to m}(i)\big)$, as proposed elsewhere [3–5]. The expectation value for the number of mutations m of the multinomial distribution is *trials* $\times$ *success probability.* In our context this means that

$$\mathbb{E}\big(\mathcal{X}_{w\to m}(i)\big) = \quad \mathcal{S}_w(i) \cdot \kappa_{w\to m}(i) \approx \quad \frac{\mathcal{R}_w(i)}{\vartheta} \cdot \kappa_{w\to m}(i) \tag{19}$$

where $\kappa_{w\to m}(i)$ denotes the probability of falsely detecting a wild type residue at position i as mutation m.

Using the multinomial model, we may correct the K^{dimer} estimation proposed in eq. (18) for the *expected noise*, from eq. (19). This yields:

$$K_m^{\text{dimer}}(i) \quad \approx \quad \frac{\mathcal{R}_w(i, \text{dimer})}{\mathcal{R}_w(i, \text{monomer})} \cdot \frac{\mathcal{R}_m(i, \text{monomer}) - \mathbb{E}\big(\mathcal{X}_{w\to m}(i, \text{monomer})\big) \cdot \vartheta(\text{monomer})}{\mathcal{R}_m(i, \text{dimer}) - \mathbb{E}\big(\mathcal{X}_{w\to m}(i, \text{monomer})\big) \cdot \vartheta(\text{dimer})} \tag{20}$$

$$= \quad \frac{\mathcal{R}_w(i, \text{dimer})}{\mathcal{R}_w(i, \text{monomer})} \cdot \frac{\mathcal{R}_m(i, \text{monomer}) - \kappa_{w\to m}(i) \cdot \mathcal{R}_w(i, \text{monomer})}{\mathcal{R}_m(i, \text{dimer}) - \kappa_{w\to m}(i) \cdot \mathcal{R}_w(i, \text{dimer})} \tag{21}$$

$$\Rightarrow \boxed{K_m^{\text{dimer}}(i) \approx \frac{\frac{\mathcal{R}_m(i,\text{monomer})}{\mathcal{R}_w(i,\text{monomer})} - \kappa_{w\to m}(i)}{\frac{\mathcal{R}_m(i,\text{dimer})}{\mathcal{R}_w(i,\text{dimer})} - \kappa_{w\to m}(i)}}, \tag{22}$$

The computation of $\kappa_{w\to m}(i)$ will be explained later in the context of the *Statistical Evaluation* (next section).

1.4 Signal-to-noise ratio

The 'signal-to-(expected)noise' ratio for each nucleotide position i and each mutant m can be computed according to:

$$D_m(i) = \frac{\mathcal{R}_m(i)}{\mathbb{E}\big(\mathcal{X}_{w\to m}(i)\big)\cdot\vartheta} \approx \frac{\mathcal{R}_m(i)}{\mathcal{R}_w(i)\cdot\kappa_{w\to m}(i)} \tag{23}$$

For all analyses, we only evaluates data where the signal-to-noise ratio is above a user-defined threshold as stated in the main manuscript (see also Fig. 1).

2 Statistical Evaluation

Note, that the above described procedure yields one *point estimate* for the effect of each mutation m at each nucleotide position i on dimerisation affinity. In the following, we describe our non-parametric method to infer the statistical certainty of the relative K^{dimer} estimates. This method is based on a leave-some-out re-sampling technique (akin to a jacknife-procedure). The re-sampling procedure will allow us to estimate the *probability distribution* of each relative K^{dimer} estimate. In order to accommodate the re-sampling procedure, we extend the previous mathematical framework.

2.1 Re-sampling Procedure

The basic idea is to determine dimerisation affinities $K^{\text{dimer}}(i, j)$ for each combination of residues (i, j), where the first residue i is mutated and the second residue j is in the wild type configuration (thus having no effect on the *relative* K^{dimer} estimate). This allows to re-estimate the effect of a mutation m at position i N-times, i.e. for each i, we can go through all $j \neq i$ (all pairs of residues), see Fig. 3 (left panel).

Since less sequence fragments will cover both i and j the further j lies away from i (see Fig. 3, left panel), the procedure is a leave-some-out re-sampling procedure in which a re-estimation is performed each time after removing a random sample from the data. The re-sampling will then give a non-parametric and unbiased probability distribution of the estimate; –in our case $K_m^{\text{dimer}}(i)$.

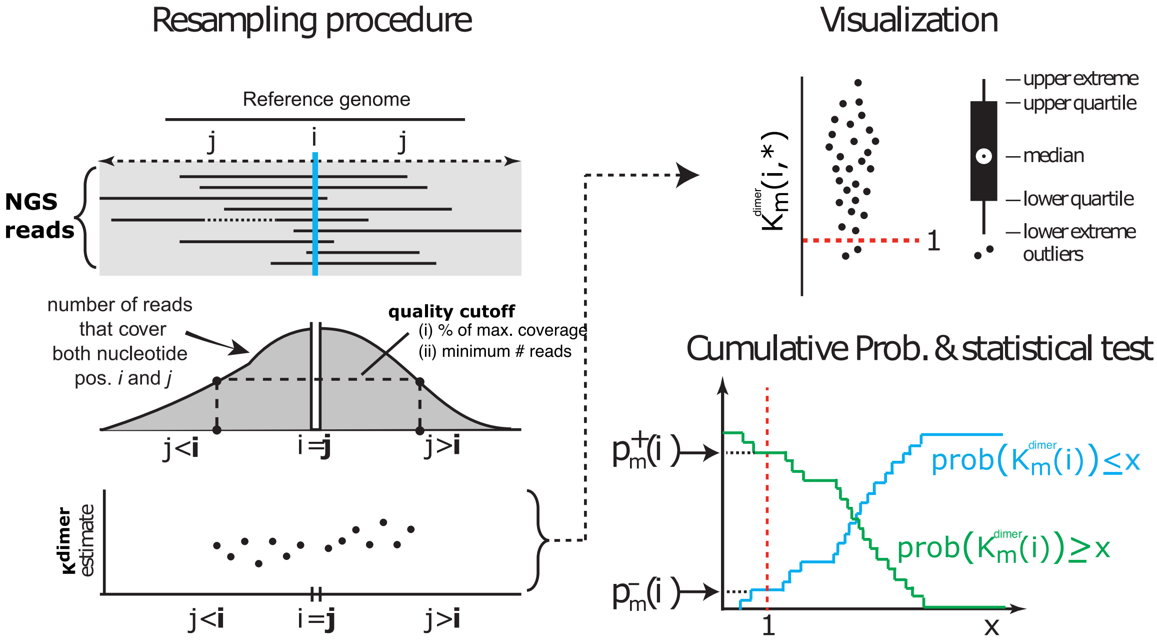

Figure 3. **Left:** Schematic of re-sampling procedure. In order to re-sample the relative dimerisation affinity $K_m^{\text{dimer}}(i)$ for mutation m and nucleotide position i, values are computed for pairs of positions (i, j), where position i is mutated and position j is wild type. Each pair of positions (i, j) yields an affinity estimate (bottom). Since the number of sequence reads that span position i and j decreases as the distance between i and j increases (middle), this is analogous to a jacknife procedure. **Right:** The re-sampling distribution for $K_m^{\text{dimer}}(i)$ can be visualized (top) and non-parametric tests (bottom) can be performed in order to detect whether a mutation m at position i significantly increases binding (with p-value: $p_m^+(i)$) or whether it significantly decreases binding in relation to the wild type (with $p_m^-(i)$)

Analogous to the previous section, **for each pair** (i,j), we get:

$$K^{\text{dimer}}_{m,w}(i,j) = \frac{\text{Kd}_{m,w}}{\text{Kd}_{w,w}}(i,j) = \frac{\mathcal{S}_{w,w}(i,j,\text{dimer})}{\mathcal{S}_{m,w}(i,j,\text{dimer})} \cdot \frac{\mathcal{S}_{m,w}(i,j,\text{monomer})}{\mathcal{S}_{w,w}(i,j,\text{monomer})}, \tag{24}$$

where $K^{\text{dimer}}_{m,w}(i,j)$ is a re-sampling estimate of $K^{\text{dimer}}_{m}(i)$, given the pair of positions (i,j).

In order to accommodate the re-sampling method we will have to extend the previously described method. We will follow the presentation as before. As in the previous section, the number of dimer/monomer sequences $\mathcal{S}$ are not known and we have the number of NGS reads $\mathcal{R}$ instead, which are subject to errors $\mathcal{X}$ (sequencing and RT-errors). In order to account for this fact, we have to update the relation between 'reads' and 'sequence numbers' for pairs of residues (i,j).

2.2 Relation between 'Reads' and 'Sequence Numbers' for re-sampling relative Kd values

The number of NGS reads $\mathcal{R}_{m_1,w}(i,j)$ with a mutation in the first site and a wild type in the second m_1, w is related to the RNA sequence numbers after protein capturing $\mathcal{S}_{m_1,w}(i,j)$ via

$$\begin{aligned}\mathcal{R}_{m_1,w}(i,j) \;=\; & \vartheta\Bigg(\mathcal{S}_{m_1,w}(i,j) - \\ & \sum_{n_1\neq m_1} \mathcal{X}_{m_1\to n_1,w}(i,j) - \sum_{m_2\in\mathbb{M}_2} \mathcal{X}_{m_1,w\to m_2}(i,j) - \sum_{n_1\neq m_1}\sum_{m_2\in\mathbb{M}_2} \mathcal{X}_{m_1\to n_1,w\to m_2}(i,j) \\ & + \sum_{n_1\neq m_1} \mathcal{X}_{n_1\to m_1,w}(i,j) + \sum_{n_2\neq w} \mathcal{X}_{m_1,n_2\to w}(i,j) + \sum_{n_1\neq m_1}\sum_{n_2\neq w} \mathcal{X}_{n_1\to m_1,n_2\to w}(i,j)\Bigg),\end{aligned} \tag{25}$$

where e.g. $\mathcal{X}_{m_1\to n_1,w}(i,j)$ denotes the number of sequences with the mutant nucleotide pair $(i = m_1, j = w)$ where the first position i was randomly detected as some other nucleotide $(m_1 \to n_1)$. In analogy with section 1.2, we can assume $\sum_{n_1\neq m_1} \mathcal{X}_{m_1\to n_1,w} \ll \mathcal{X}_{w\to m_1,w} \gg \mathcal{X}_{k_1\to m_1,w}$. Also, since double mutated sequences are assumed to be much less abundant than single mutated sequences, we assume $\sum_{m_2\in\mathbb{M}_2} \mathcal{X}_{m_1,w\to m_2}(i,j) \gg \sum_{n_1\neq m_1}\sum_{m_2\in\mathbb{M}_2} \mathcal{X}_{m_1\to n_1,w\to m_2}(i,j)$ and $\sum_{n_2\neq w} \mathcal{X}_{m_1,n_2\to w}(i,j) \gg \sum_{n_1\neq m_1}\sum_{n_2\neq w} \mathcal{X}_{n_1\to m_1,n_2\to w}(i,j)$. Therefore, eq. (25) simplifies to:

$$\begin{aligned}\mathcal{R}_{m_1,w}(i,j) \;&\approx\; \vartheta\Bigg(\mathcal{S}_{m_1,w}(i,j) + \mathcal{X}_{w\to m_1,w}(i,j) + \sum_{m_2\in\mathbb{M}_2} \mathcal{X}_{m_1,w\to m_2}(i,j) + \sum_{n_2\neq w} \mathcal{X}_{m_1,n_2\to w}(i,j)\Bigg), \\ &\approx\; \vartheta\Bigg(\mathcal{S}_{m_1,w}(i,j) + \mathcal{X}_{w\to m_1,w}(i,j)\Bigg),\end{aligned} \tag{26}$$

where the last step is motivated by the fact that the mutation rate in the library is usually low and thus RNAs carrying a mutation at either i or j are less abundant than the double wild type. From here we get

$$\mathcal{S}_{m_1,w}(i,j) \;\approx\; \frac{\mathcal{R}_{m_1,w}(i,j)}{\vartheta} - \mathcal{X}_{w\to m_1,w}(i,j). \tag{27}$$

In analogy, we get

$$\begin{aligned}\mathcal{S}_{w,w}(i,j) \;&\approx\; \frac{\mathcal{R}_{w,w}(i,j)}{\vartheta} + \sum_{m_2\in\mathbb{M}_2} \mathcal{X}_{w,w\to m_2}(i,j) + \sum_{m_1\in\mathbb{M}_1} \mathcal{X}_{w\to m_1,w}(i,j) \\ \mathcal{S}_{w,w}(i,j) \;&\approx\; \frac{\mathcal{R}_{w,w}(i,j)}{\vartheta}.\end{aligned} \tag{28}$$

Combining the equations (27) and (28) with eq. (24), we get

$$K_{m_1,w}^{\text{dimer}}(i,j) \approx \frac{\mathcal{R}_{w,w}(i,j,\text{dimer})}{\mathcal{R}_{w,w}(i,j,\text{monomer})} \cdot \frac{\mathcal{R}_{m_1,w}(i,j,\text{monomer}) - \vartheta(\text{monomer}) \cdot \mathcal{X}_{w\to m_1,w}(i,j,\text{monomer})}{\mathcal{R}_{m_1,w}(i,j,\text{dimer}) - \vartheta(\text{dimer}) \cdot \mathcal{X}_{w\to m_1,w}(i,j,\text{dimer})}, \tag{29}$$

which is in analogy to eq. (18).

2.3 Error correction

Once again, we assume that the *noise* $\mathcal{X}$ is multinomially distributed, i.e.:
$\mathcal{X}_{w\to m_1,w}(i,j) \sim \mathcal{M}\big(\mathcal{S}_{w,w}(i,j), \kappa_{w\to m_1,w}(i,j)\big)$. In our context this means that

$$\mathbb{E}\big(\mathcal{X}_{w\to m_1,w}(i,j)\big) = \quad \mathcal{S}_{w,w}(i,j) \cdot \kappa_{w\to m_1,w}(i,j) \approx \quad \frac{\mathcal{R}_{w,w}(i,j)}{\vartheta} \cdot \kappa_{w\to m_1,w}(i,j) \tag{30}$$

Correspondingly we get,

$$K_{m_1,w}^{\text{dimer}}(i,j) \approx \frac{\frac{\mathcal{R}_{m_1,w}(i,j,\text{monomer})}{\mathcal{R}_{w,w}(i,j,\text{monomer})} - \kappa_{w\to m_1,w}(i,j)}{\frac{\mathcal{R}_{m_1,w}(i,j,\text{dimer})}{\mathcal{R}_{w,w}(i,j,\text{dimer})} - \kappa_{w\to m_1,w}(i,j)} \tag{31}$$

$$\boxed{K_{m_1,w}^{\text{dimer}}(i,j) \approx \frac{\frac{\mathcal{R}_{m_1,w}(i,j,\text{monomer})}{\mathcal{R}_{w,w}(i,j,\text{monomer})} - \mathbb{E}_J\big(\kappa_{w\to m_1}(i)\big)}{\frac{\mathcal{R}_{m_1,w}(i,j,\text{dimer})}{\mathcal{R}_{w,w}(i,j,\text{dimer})} - \mathbb{E}_J\big(\kappa_{w\to m_1}(i)\big)}} \tag{32}$$

which can be used to re-compute $K_{m_1}^{\text{dimer}}(i)$.

2.4 Signal-to-noise ratio

The 'signal-to-(expected)noise' ratio for mutant nucleotide position i and co-varying wild type position j can be computed according to:

$$\begin{aligned} D_{m_1,w}(i,j) &= \frac{\mathcal{R}_{m_1,w}(i,j)}{\mathbb{E}\big(\mathcal{X}_{w\to m_1,w}(i,j)\big)\cdot\vartheta} \approx \frac{\mathcal{R}_{m_1,w}(i,j)}{\mathcal{R}_{w,w}(i,j)\cdot \kappa_{w\to m_1,w}(i,j)} \\ &\approx \frac{\mathcal{R}_{m_1,w}(i,j)}{\mathcal{R}_{w,w}(i,j)\cdot \mathbb{E}_J\big(\kappa_{w\to m_1}(i)\big)} \end{aligned} \tag{33}$$

For all analyses, we only evaluate data where the signal-to-noise ratio was above the threshold defined in the main manuscript.

2.5 Estimation of error probability κ

If experiments with non-mutated RNA are conducted in parallel (see also Fig. 1), we have $\mathcal{S}_{w,m_2}(i,j) = \mathcal{S}_{m_1,w}(i,j) = \mathcal{S}_{m_1,m_2}(i,j) = 0$ and can thus estimate the probability of falsely detecting a wild type residue at position i as some mutant. In fact, the re-sampling scheme allows us to compute statistical properties of the error probability

$$\mathbb{E}_J\big(\kappa_{w\to m_1}(i)\big) \quad \approx \quad N^{-1} \cdot \sum_{j\in J} \frac{\mathcal{R}_{m_1,w}(i,j)}{\mathcal{R}_{w,w}(i,j)} \tag{34}$$

where N denotes the number of co-varying positions $j \in J$ that have sufficient read coverage. Thus, along the same lines as the re-sampling scheme for the relative dimerisation affinity, we can estimate a confidence range for the error probability $\kappa_{w\to m}(i)$.

2.6 Quality criteria

1. In order to statistically evaluate whether a mutation m at position i significantly affects binding, we first apply eq. (32) for all position $j \in J$ that have a sufficient signal-to-noise ratio (see eq. (33)). "Sufficient" is defined as follows: If the ratio is below a user-supplied threshold both in the dimer and monomer samples, the corresponding Kd estimate $K^{\text{dimer}}_{m_1,w}(i,j)$ is discarded. If the signal is below the threshold at either the dimer- or monomer samples, the respective estimate is tagged as either being a lower- or upper estimate of $K^{\text{dimer}}_{m_1,w}(i,j)$ and assigned (imputed) the value of the median $K^{\text{dimer}}_{m_1,w}(i,*)$ estimate. This has the following reason: If a mutation strongly increases dimerisation affinity, all sequences carrying this mutation may be dimerised, and none- or too little amounts of sequence may remain monomeric. Thus, $K^{\text{dimer}}_{m_1,w}(i,j)$ may not be accurately determined and we can assume that $K^{\text{dimer}}_{m_1,w}(i,j)$ may in fact be lower than estimable.

2. For the re-sampling procedure as illustrated in Fig. 3 (left), only positions j are evaluated where the total number of sequence fragments covering both i and j has at least a user-defined 50 % of the maximum coverage (middle panel in Fig. 3, left). Secondly, the total number of reads that have to be available (middle panel in Fig. 3, left) has to exceed a value stated by the user ('minimum coverage criterium'). Both criteria together ensure that each re-sampling is based on a sufficient number of reads and thus provides meaningful estimates.

3. The $\text{K}_{m_1,w}(i,j)$ values then give rise to an empirical distribution (see Fig. 3, left (lower panel) and right (upper panel)). A minimum number of re-samplings is required in order to reconstruct the empirical distribution with sufficient confidence (see Fig. 3, lower panel on the right).

2.7 Statistical test

The statistical test can subsequently be performed on the re-sampling distribution, see Fig. 3 (lower panel on the right): To test whether a mutation at position i significantly increases K^{dimer} (i.e. decreases dimerization), i.e. $\mathcal{H}_0 : K^{\text{dimer}}_m(i) \leq 1$, $\mathcal{H}_1 : K^{\text{dimer}}_m(i) > 1$, the raw p-value (= probability of type I error/false rejection of the null hypothesis) can be computed according to:

$$p^-_m(i) \quad = \quad \frac{\# K^{\text{dimer}}_{m,w}(i,*) \leq 1}{\# K^{\text{dimer}}_{m,w}(i,*)}, \tag{35}$$

where # denotes the 'number of estimates' and '*' indicates that all N positions $j \in J$ are evaluated that pass the quality criteria (previous section). To test if mutation m at position i increases dimerization, the p-value is calculated according to:

$$p^+_m(i) \quad = \quad \frac{\# K^{\text{dimer}}_{m,w}(i,*) \geq 1}{\# K^{\text{dimer}}_{m,w}(i,*)}. \tag{36}$$

When several nucleotide positions i are assessed, test corrections need to be performed. All p-values reported are corrected by Benjamini-Hochberg false discovery rate method (BHFDR) [6], which proceeds as follows: All p-values are sorted in ascending order and p-values are corrected according to

$$\tilde{p}_k = p_k \cdot K/k \tag{37}$$

where p_k and $\tilde{p}_k$ denote the k-smallest raw- and corrected p-value and K denotes the total number of p-values computed.
There is a significant impact of mutation m at nucleotide position i, if $\tilde{p} < \alpha$.
Note: The significance level α (prob. of type I error/*false positive*) is set to $\alpha = 0.05$ in all analyses performed in the main manuscript.

3 Dimerisation of compensatory mutants

The effect that a mutation has on dimerisation is derived under the assumption that the second RNA, with which the mutant RNA forms a dimer, carries a wildtype conformation at the corresponding binding position.
In theory, the calculated effects of mutations that decrease dimerisation could be perturbed by pairing with binding partners which have a compensatory mutation at the particular binding site, and thus would have a 'wildtype-like' binding affinity. However, the likelihood for this to happen is negligible as outlined below, and would thus have no impact on the quantitative estimation of the binding affinity of the mutant.

In the main manuscript (first section of the results), we observe mutation frequencies of $5.4 \times 10^{-3} \approx 0.005$ in the mutant libraries. Hence, a mutation at a dimerisation-affecting site occurs with probability $p_1 \approx 0.005$. Likewise, the probability of a compensatory mutation is $p_2 \approx 0.005$, or even less since in most cases only one of the three possible mutations would redeem the negative effect of the mutation (e.g. 'A:U' Watson-Crick interaction mutating to 'C:G'). In contrast, the probability that a wildtype, or no compensatory mutation occurs in the RNA is $p_3 \approx 1 - 0.005 = 0.995$.
Consequently, in the pool of RNA, the signal that relates to dimerisation with the wildtype is at least $\frac{p_1 \cdot p_3}{p_1 \cdot p_2} = \frac{0.005 \cdot 0.995}{0.005 \cdot 0.005} = 199$ times stronger than the signal that relates to the dimerisation of two compensatory mutant RNAs.

This argument is supported by the results of our study, showing the strongest dimerisation signal for the 6-nucleotide palindromic sequence, where many trans-complementary mutations are described in the literature [7, 8]. We therefore disregard the potential, but virtually non-existent noise that could be introduced by compensatory mutant dimers.

References

1. Smyth RP, Despons L, Huili G, Bernacchi S, Hijnen M, et al. (2015) Mutational interference mapping experiment (MIME) for studying rna structure and function. Nat Methods 12: 866–872.

2. Smith MR, Smyth RP, Marquet R, von Kleist M (2016) MIMEAnTo: profiling functional rna in mutational interference mapping experiments. Bioinformatics 32: 3369-3370.

3. Zagordi O, Bhattacharya A, Eriksson N, Beerenwinkel N (2011) ShoRAH: estimating the genetic diversity of a mixed sample from next-generation sequencing data. BMC Bioinformatics 12: 119.

4. Prosperi MCF, Prosperi L, Bruselles A, Abbate I, Rozera G, et al. (2011) Combinatorial analysis and algorithms for quasispecies reconstruction using next-generation sequencing. BMC Bioinformatics 12: 5.

5. Prabhakaran S, Rey M, Zagordi O, Beerenwinkel N, Roth V (2013) HIV haplotype inference using a propagating dirichlet process mixture model. IEEE/ACM Trans Comput Biol Bioinform .

6. Benjamini Y, Hochberg Y (1995) Controlling the false discovery rate: a practical and powerful approach to multiple testing. Journal of the Royal Statistical Society Series B Methodological 57: 289–300.

7. Paillart JC, Shehu-Xhilaga M, Marquet R, Mak J (2004) Dimerization of retroviral RNA genomes: an inseparable pair. Nature Reviews Microbiology 2: 461–472.

8. Dubois N, Marquet R, Paillart JC, Bernacchi S (2018) Retroviral RNA dimerization: from structure to functions. Frontiers in microbiology 9: 527.
