# Supplementary material for: Short- and long-range interactions in the HIV-1 5′ UTR regulate genome dimerization and packaging
Source: Nat Struct Mol Biol. 2022 Mar 28;29(4):306–19. doi: 10.1038/s41594-022-00746-2 (PMC9010304; doi:10.1038/s41594-022-00746-2)
Supplement: Supplementary file 2 — Reporting Summary [file 41594_2022_746_MOESM2_ESM.pdf]

nature portfolio

Corresponding author(s): Redmond P. Smyth

Last updated by author(s): Feb 1, 2022

Reporting Summary

Nature Portfolio wishes to improve the reproducibility of the work that we publish. This form provides structure for consistency and transparency in reporting. For further information on Nature Portfolio policies, see our Editorial Policies and the Editorial Policy Checklist.

Statistics

For all statistical analyses, confirm that the following items are present in the figure legend, table legend, main text, or Methods section.

n/a	Confirmed	
☐	☒	The exact sample size (*n*) for each experimental group/condition, given as a discrete number and unit of measurement
☐	☒	A statement on whether measurements were taken from distinct samples or whether the same sample was measured repeatedly
☐	☒	The statistical test(s) used AND whether they are one- or two-sided<br>*Only common tests should be described solely by name; describe more complex techniques in the Methods section.*
☒	☐	A description of all covariates tested
☐	☒	A description of any assumptions or corrections, such as tests of normality and adjustment for multiple comparisons
☐	☒	A full description of the statistical parameters including central tendency (e.g. means) or other basic estimates (e.g. regression coefficient) AND variation (e.g. standard deviation) or associated estimates of uncertainty (e.g. confidence intervals)
☐	☒	For null hypothesis testing, the test statistic (e.g. *F*, *t*, *r*) with confidence intervals, effect sizes, degrees of freedom and *P* value noted<br>*Give P values as exact values whenever suitable.*
☒	☐	For Bayesian analysis, information on the choice of priors and Markov chain Monte Carlo settings
☒	☐	For hierarchical and complex designs, identification of the appropriate level for tests and full reporting of outcomes
☒	☐	Estimates of effect sizes (e.g. Cohen's *d*, Pearson's *r*), indicating how they were calculated

Our web collection on statistics for biologists contains articles on many of the points above.

Software and code

Policy information about availability of computer code

Data collection	Data was generated on commerical Illumina sequencing (Nextseq, Novaseq and miniseq) and Nanotemper microscale thermophoresis platforms
Data analysis	Data was analysed using readily available software tools. Mutational interference data was analysed using MIMEAnTo (10.1093/bioinformatics/btw47). RNA structure data was analysed using ShapeMapper2 (10.1261/rna.061945.117), Vienna RNA 2.0 package (10.1186/1748-7188-6-26), RNA Framework (10.1007/978-1-0716-1307-8_5), and a modified deltaSHAPE analysis (10.1021/acs.biochem.5b00977). Multi-dimensional RNA structural probing data was analysed using the methods outlined in 10.1073/pnas.1619897114. Bootstrapping was performed using rna_structure function of the Basic Inference Engine for RNA structure (https://ribokit.github.io/Biers/) (MST data were processed using MO Affinity Analysis software (v 2.3; NanoTemper 565 Technologies). MST graphs were plotted using GraphPad Prism 8.4.3 software. Visualisations we made using python scikit-learn library (v 0.23.2), python NumPy library (v 1.19.2), python seaborn library (v 0.11.1) and python matplotlib library (v 3.3.2).

For manuscripts utilizing custom algorithms or software that are central to the research but not yet described in published literature, software must be made available to editors and reviewers. We strongly encourage code deposition in a community repository (e.g. GitHub). See the Nature Portfolio guidelines for submitting code & software for further information.

Data

Policy information about availability of data

All manuscripts must include a data availability statement. This statement should provide the following information, where applicable:

- Accession codes, unique identifiers, or web links for publicly available datasets
- A description of any restrictions on data availability
- For clinical datasets or third party data, please ensure that the statement adheres to our policy

Raw sequencing data are accessible through NCBI bioproject id PRJNA771368. HIV-1 sequences were downloaded from the Los Alamos HIV-1 sequence database (https://www.hiv.lanl.gov/content/index). In the main manuscript, conclusions are drawn from pooled data, but unpooled data are provided as extensive supplementary data. Pooled data is grouped into 4 structural classes. Unpooled data comprises 24 independently generated samples. DMS reactivities and Kdimer measurements Specifically, unpooled Kdimer and DMS reactivities are provided in table form. Also, for unpooled data, Kdimer and DMS reactivities are mapped to predicted dimer, predicted monomer, refined dimer and refined monomer strucrures. Structural predictions derived from DMS reactivities from unpooled data sets are provided as svg files. For two dimensional analysis, for each stage of the analysis (mutation rates, z-scores, convolution filtered data, detected helix and best helixes) are provided as pdf files.

Field-specific reporting

Please select the one below that is the best fit for your research. If you are not sure, read the appropriate sections before making your selection.

☒ Life sciences ☐ Behavioural & social sciences ☐ Ecological, evolutionary & environmental sciences

For a reference copy of the document with all sections, see nature.com/documents/nr-reporting-summary-flat.pdf

Life sciences study design

All studies must disclose on these points even when the disclosure is negative.

Sample size	No statistical method was used to predetermine sample size. Data from 24 independent samples were pooled into 4 distinct structural classes for analysis. RNA-seq experiments to measure cellular packaging efficiencies were performed in duplicate.
Data exclusions	No data were excluded from the analysis
Replication	Conclusions were verified using gel based assays, replicated at a minimum twice
Randomization	Randomization was not performed. Rather, sample pooling was performed based on a PCA analysis.
Blinding	Blinding was not performed

Reporting for specific materials, systems and methods

We require information from authors about some types of materials, experimental systems and methods used in many studies. Here, indicate whether each material, system or method listed is relevant to your study. If you are not sure if a list item applies to your research, read the appropriate section before selecting a response.

Materials & experimental systems

n/a	Involved in the study	
☒	☐	Antibodies
☐	☒	Eukaryotic cell lines
☒	☐	Palaeontology and archaeology
☒	☐	Animals and other organisms
☒	☐	Human research participants
☒	☐	Clinical data
☒	☐	Dual use research of concern

Methods

n/a	Involved in the study	
☒	☐	ChIP-seq
☒	☐	Flow cytometry
☒	☐	MRI-based neuroimaging

Eukaryotic cell lines

Policy information about cell lines

Cell line source(s)	293T cells, a gift from the Caliskan laboratory
Authentication	293T cells were not verified

Mycoplasma contamination	293T cell lines is regularly tested for mycoplasm infection (monthly)
Commonly misidentified lines (See ICLAC register)	N/A
