# Supplementary material for: Short- and long-range interactions in the HIV-1 5′ UTR regulate genome dimerization and packaging
Source: Nat Struct Mol Biol. 2022 Mar 28;29(4):306–19. doi: 10.1038/s41594-022-00746-2 (PMC9010304; doi:10.1038/s41594-022-00746-2)
Supplement: Supplementary file 6 — Conservation of predicted interactions. [file 41594_2022_746_MOESM6_ESM.zip › Conservation/Conservation.pdf]

Conservation

The conservation of these polyA-SL1 and PBS-SL1 interactions were assess using approximately 800 curated sequences downloaded from the Los Alamos HIV sequence database.

1. polyA-SL1 interaction

polyA sequence conservation, modal sequence

Modal Sequence: 5'																		
position	80	81	82	83	84	85	86	87	88	89	90	91	92	93	94	95	96	97
base	C	T	T	G	C	C	T	T	G	A	G	T	G	C	T	T	C	A
a	1	1	1	0	0	0	0	0	9	887	0	0	6	0	7	20	76	867
u	0	890	889	1	0	5	877	885	0	0	0	884	0	3	860	804	308	0
c	890	0	0	0	887	883	6	1	1	0	0	0	0	884	1	46	485	1
g	0	0	1	889	0	0	0	1	877	0	889	3	881	0	3	1	2	0
count	890	890	889	889	887	883	877	885	877	887	889	884	881	884	860	804	485	867
total	891	891	891	890	887	888	883	887	887	887	889	887	887	887	871	871	871	868
modal conservation	99%	99%	99%	99%	100%	99%	99%	99%	98%	100%	100%	99%	99%	99%	98%	92%	55%	99%

SL1 sequence conservation, modal sequence

Modal Sequence: 3'																		
position	253	254	255	256	257	258	259	260	261	262	263	264	265	266	267	268	269	270
base	T	G	A	A	G	C	G	C	G	C	A	C	G	G	C	A	A	G
a	0	2	651	687	9	0	0	0	5	0	653	0	170	5	0	685	685	3
u	688	0	2	0	1	15	0	2	0	0	6	4	0	0	0	0	0	0
c	0	0	1	0	7	667	0	685	0	684	1	681	4	3	685	0	0	0
g	0	684	0	0	664	0	687	0	681	0	24	0	511	677	0	0	0	682
count	688	684	651	687	664	667	687	685	681	684	653	681	511	677	685	685	685	682
total	688	686	654	687	681	682	687	687	686	684	684	685	685	685	685	685	685	685
modal conservation	100%	99%	99%	100%	97%	97%	100%	99%	99%	100%	95%	99%	74%	98%	100%	100%	100%	99%

SL1-polyA interaction conservation

<table>
<tr><th colspan="19">Conservation of Interaction</th></tr>
<tr><td>position1</td><td>80</td><td>81</td><td>82</td><td>83</td><td>84</td><td>85</td><td>86</td><td>87</td><td>88</td><td>89</td><td>90</td><td>91</td><td>92</td><td>93</td><td>94</td><td>95</td><td>96</td><td>97</td></tr>
<tr><td>base1</td><td>C</td><td>T</td><td>T</td><td>G</td><td>C</td><td>C</td><td>T</td><td>T</td><td>G</td><td>A</td><td>G</td><td>T</td><td>G</td><td>C</td><td>T</td><td>T</td><td>C</td><td>A</td></tr>
<tr><td>interaction</td><td>|</td><td>|</td><td>|</td><td>|</td><td>|</td><td>|</td><td>-</td><td>|</td><td>|</td><td>-</td><td>|</td><td>|</td><td>|</td><td>|</td><td>|</td><td>|</td><td>|</td><td>|</td></tr>
<tr><td>base2</td><td>G</td><td>A</td><td>A</td><td>C</td><td>G</td><td>G</td><td>C</td><td>A</td><td>C</td><td>G</td><td>C</td><td>G</td><td>C</td><td>G</td><td>A</td><td>A</td><td>G</td><td>T</td></tr>
<tr><td>position2</td><td>270</td><td>269</td><td>268</td><td>267</td><td>266</td><td>265</td><td>264</td><td>263</td><td>262</td><td>261</td><td>260</td><td>259</td><td>258</td><td>257</td><td>256</td><td>255</td><td>254</td><td>253</td></tr>
<tr><td>interacting</td><td>679</td><td>682</td><td>681</td><td>681</td><td>674</td><td>515</td><td>0</td><td>672</td><td>671</td><td>0</td><td>686</td><td>684</td><td>678</td><td>662</td><td>679</td><td>590</td><td>617</td><td>687</td></tr>
<tr><td>not interacting</td><td>4</td><td>1</td><td>2</td><td>1</td><td>8</td><td>168</td><td>679</td><td>9</td><td>10</td><td>684</td><td>0</td><td>1</td><td>2</td><td>17</td><td>7</td><td>63</td><td>68</td><td>0</td></tr>
<tr><td>conservation</td><td>99%</td><td>99%</td><td>99%</td><td>99%</td><td>98%</td><td>75%</td><td>0%</td><td>98%</td><td>98%</td><td>0%</td><td>100%</td><td>99%</td><td>99%</td><td>97%</td><td>98%</td><td>90%</td><td>90%</td><td>100%</td></tr>
</table>

These regions are highly conserved, with some polymorphism at positions 95/96 in the polyA. The modal sequence from this alignment differs slightly from the NL43 construct used in our study. Nevertheless, i the polymorphisms maintain the polyA-SL1 interaction (and even extend it).

There is one polymorphic position 265 in SL1. The G to A mutation does not conserve the interaction with its partner directly opposite, but this mutation is not disruptive as it can instead base pair with the unpaired U86 opposite.

2. PBS-SL1 interaction

PBS sequence conservation, modal sequence

Modal Sequence: 5'															
position	210	211	212	213	214	215	216	217	218	219	220	221	222	223	224
base	A	A	G	T	A	A	A	A	C	C	A	G	A	G	G
a	685	687	4	115	478	344	638	638	0	0	552	4	682	0	136
u	0	0	0	451	5	4	13	3	0	0	0	1	0	0	5
c	2	0	0	7	0	0	0	0	686	687	1	0	0	0	3
g	0	0	681	113	204	338	35	46	0	0	133	679	2	685	543
count	685	687	681	451	478	344	638	638	686	687	552	679	682	685	543
total	687	687	685	686	687	686	686	687	686	687	686	684	684	685	687
modal conservation	99%	100%	99%	65%	69%	50%	93%	92%	100%	100%	80%	99%	99%	100%	79%

SL1 sequence conservation, modal sequence

Modal Sequence: 3'															
position	241	242	243	244	245	246	247	248	249	250	251	252	253	254	255
base	G	A	C	T	C	G	G	C	T	T	G	C	T	G	A
a	2	685	0	0	1	0	0	0	0	0	0	0	0	2	651
u	0	0	1	688	4	2	2	0	688	688	0	2	688	0	2
c	0	0	687	0	682	0	0	688	0	0	0	682	0	0	1
g	686	3	0	0	1	686	686	0	0	0	688	3	0	684	0
count	686	685	687	688	682	686	686	688	688	688	688	682	688	684	651
total	688	688	688	688	688	688	688	688	688	688	688	687	688	686	654
modal conservation	99%	99%	99%	100%	99%	99%	99%	100%	100%	100%	100%	99%	100%	99%	99%

The SL1 sequence is almost universally conserved. We identified sequence variants in the PBS region, resulting in some structural variation, but all are predicted to form hairpins of similar stability. The figure below shows the predicted structure of the top 116, 71, 50 sequence variants.

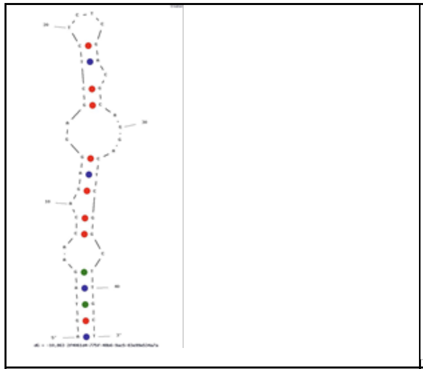

116 seq - dG = -10.863

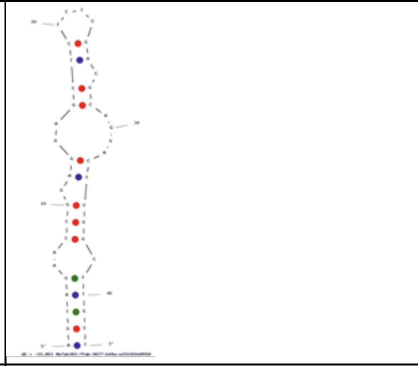

71 seq - dG = -10.863

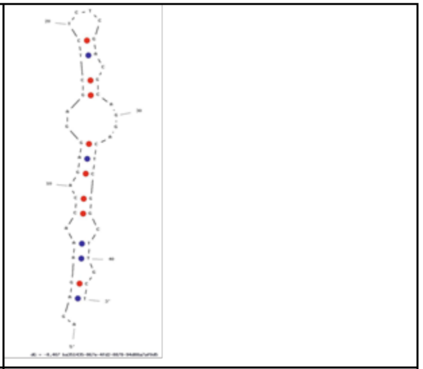

50 seq - dG = -8.407
