# Supplementary figures and images for: Short- and long-range interactions in the HIV-1 5′ UTR regulate genome dimerization and packaging
Source: Nat Struct Mol Biol. 2022 Mar 28;29(4):306–19. doi: 10.1038/s41594-022-00746-2 (PMC9010304; doi:10.1038/s41594-022-00746-2)

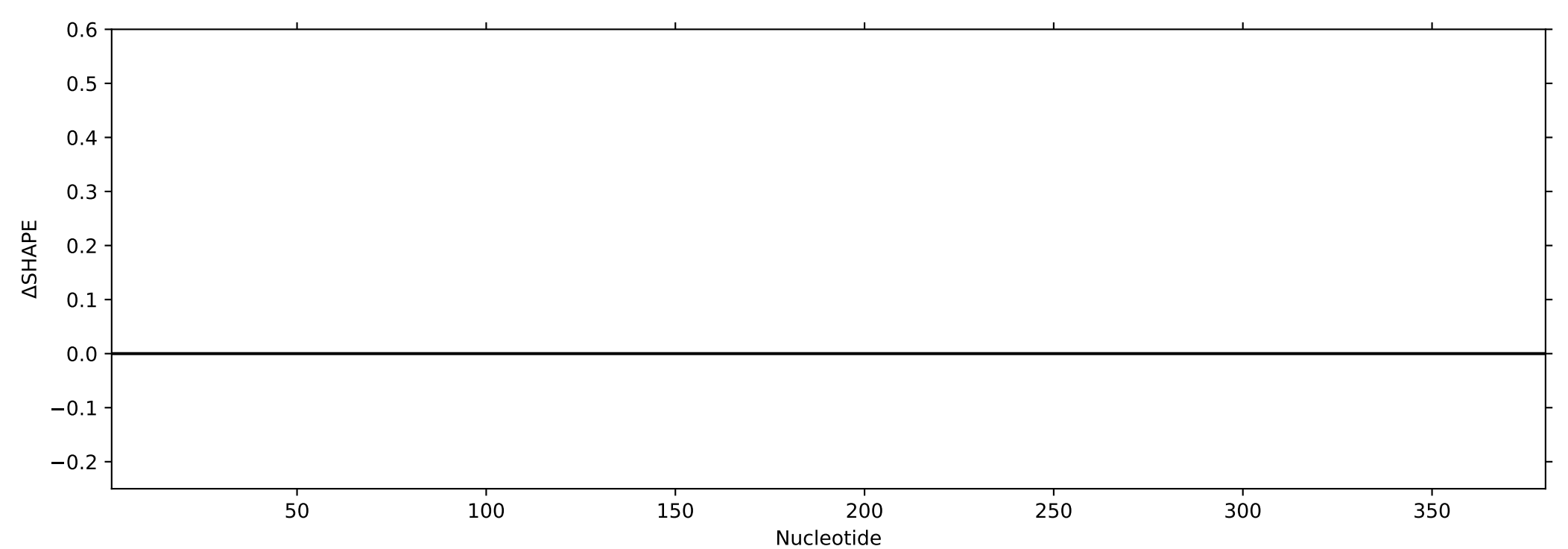

Supplement: Supplementary file 7 — Statistical deltaSHAPE analysis. [file 41594_2022_746_MOESM7_ESM.zip › deltaSHAPE/oneGtwoG_monomer_vs_oneGtwoG_monomer.pdf]

$\Delta$ SHAPE

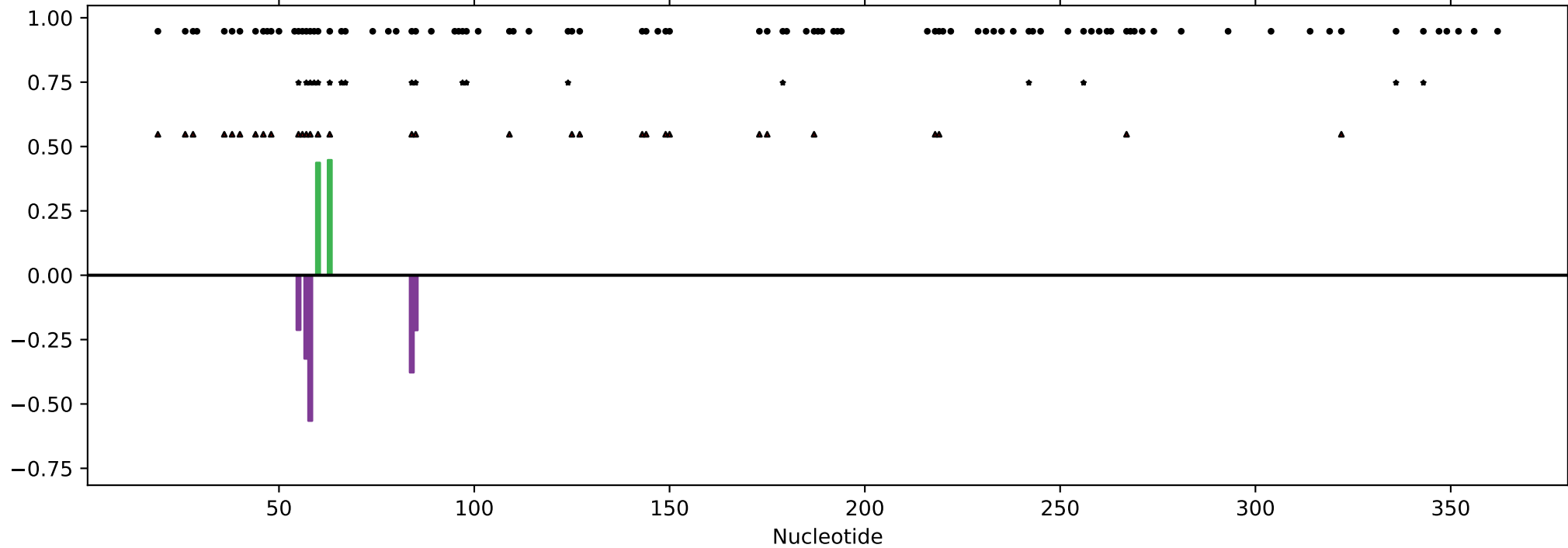

Supplement: Supplementary file 7 — Statistical deltaSHAPE analysis. [file 41594_2022_746_MOESM7_ESM.zip › deltaSHAPE/threeG_monomer_vs_oneGtwoG_monomer.pdf]

$\Delta$ SHAPE

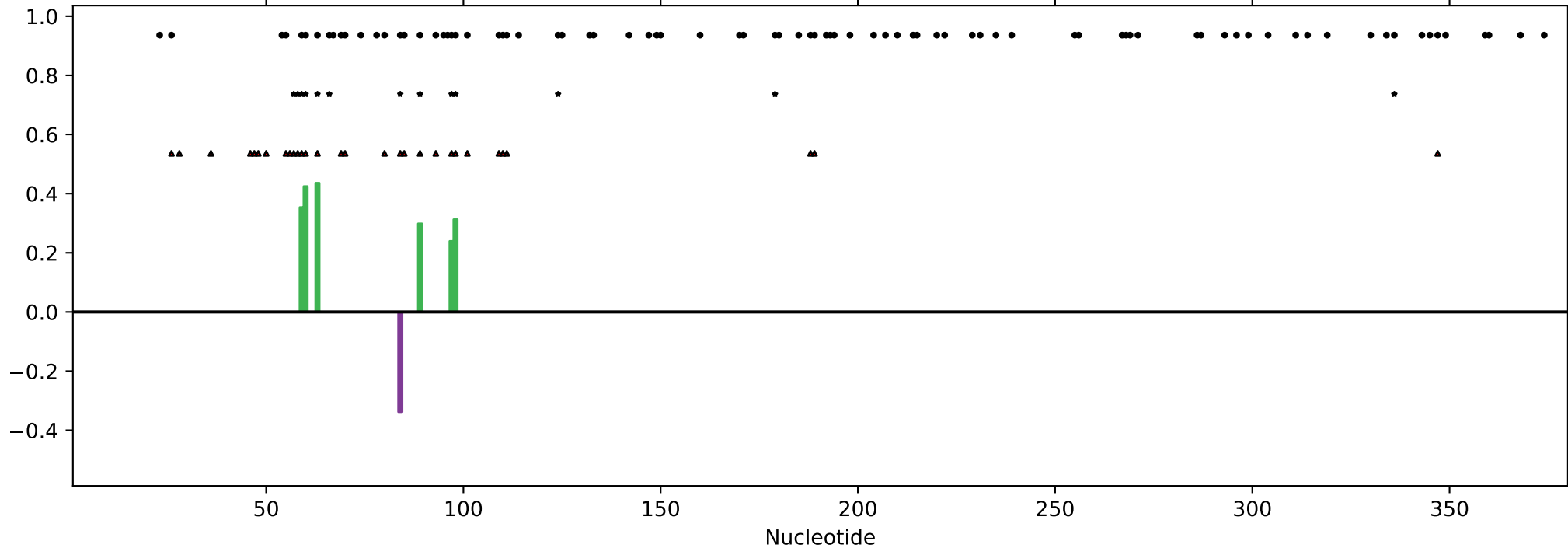

Supplement: Supplementary file 7 — Statistical deltaSHAPE analysis. [file 41594_2022_746_MOESM7_ESM.zip › deltaSHAPE/threeG_dimer_vs_oneGtwoG_dimer.pdf]

$\Delta$ SHAPE

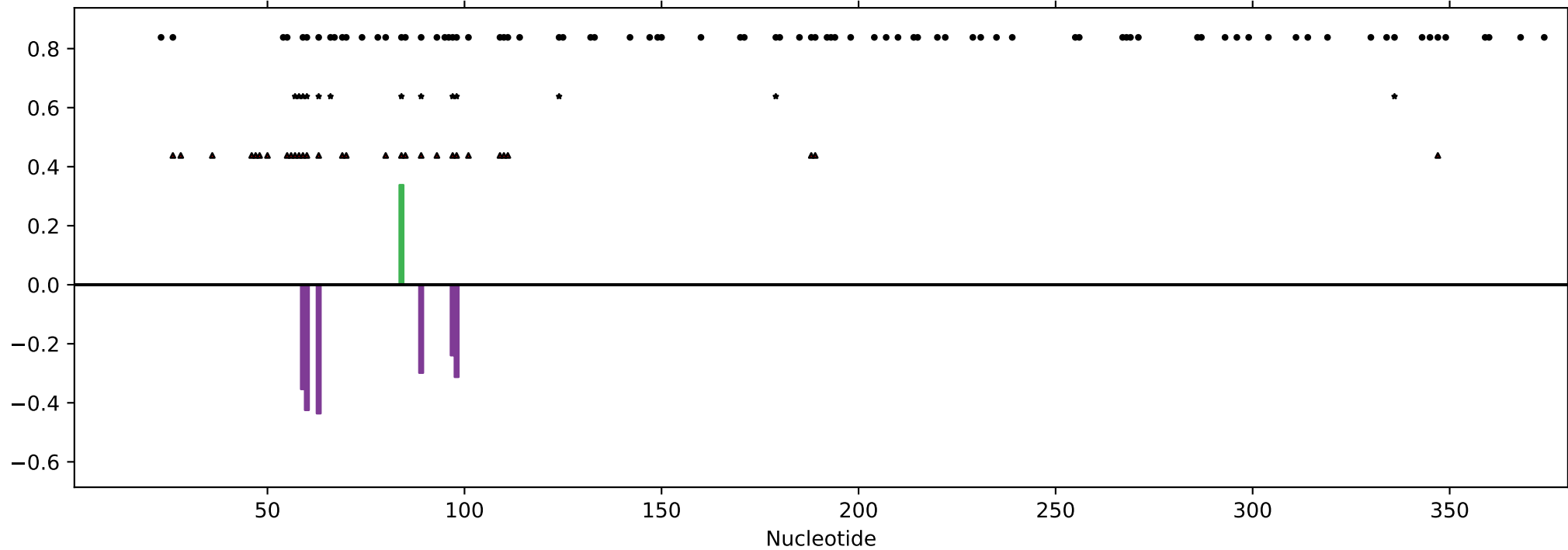

Supplement: Supplementary file 7 — Statistical deltaSHAPE analysis. [file 41594_2022_746_MOESM7_ESM.zip › deltaSHAPE/oneGtwoG_dimer_vs_threeG_dimer.pdf]

$\Delta$ SHAPE

1.00  
0.75  
0.50  
0.25  
0.00  
-0.25  
-0.50  
-0.75

Nucleotide

50

100

150

200

250

300

350

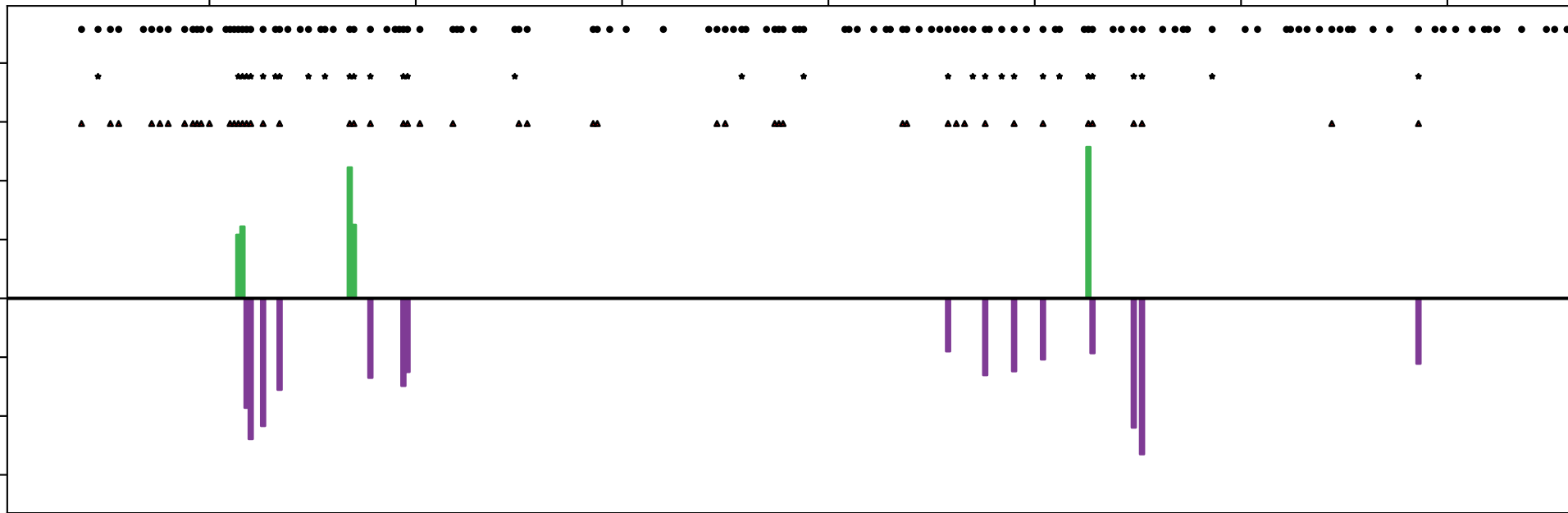

Supplement: Supplementary file 7 — Statistical deltaSHAPE analysis. [file 41594_2022_746_MOESM7_ESM.zip › deltaSHAPE/oneGtwoG_dimer_vs_threeG_monomer.pdf]

$\Delta$ SHAPE

1.00  
0.75  
0.50  
0.25  
0.00  
-0.25  
-0.50  
-0.75

Nucleotide

50

100

150

200

250

300

350

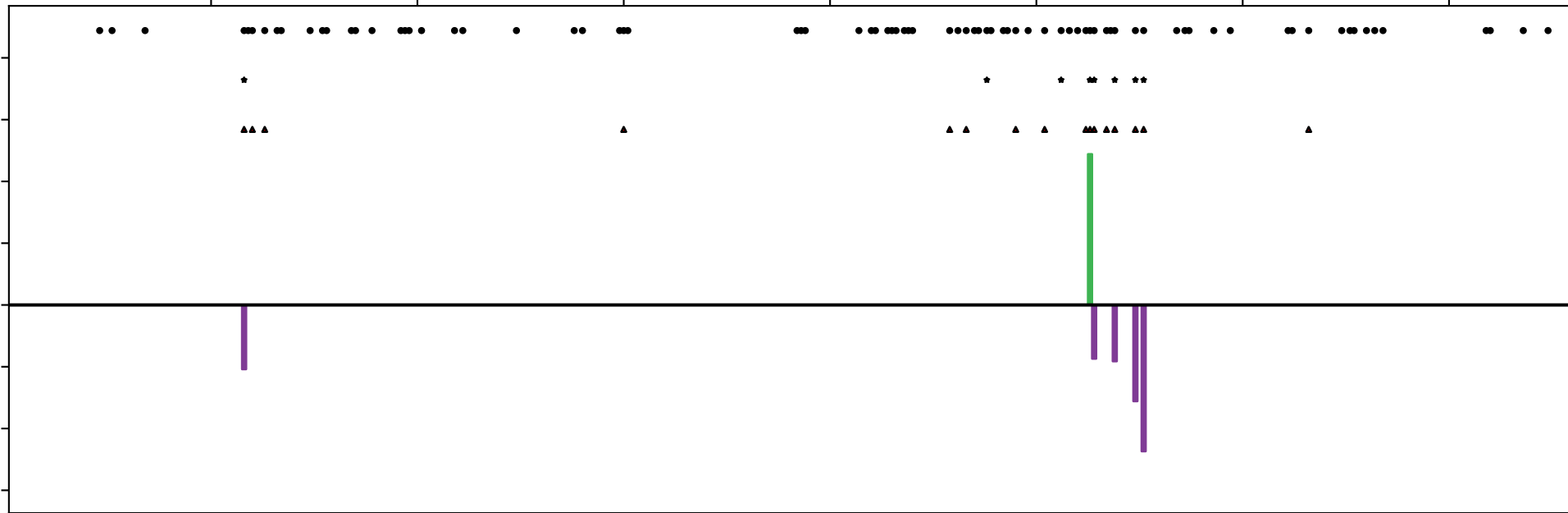

Supplement: Supplementary file 7 — Statistical deltaSHAPE analysis. [file 41594_2022_746_MOESM7_ESM.zip › deltaSHAPE/oneGtwoG_dimer_vs_oneGtwoG_monomer.pdf]

$\Delta$ SHAPE

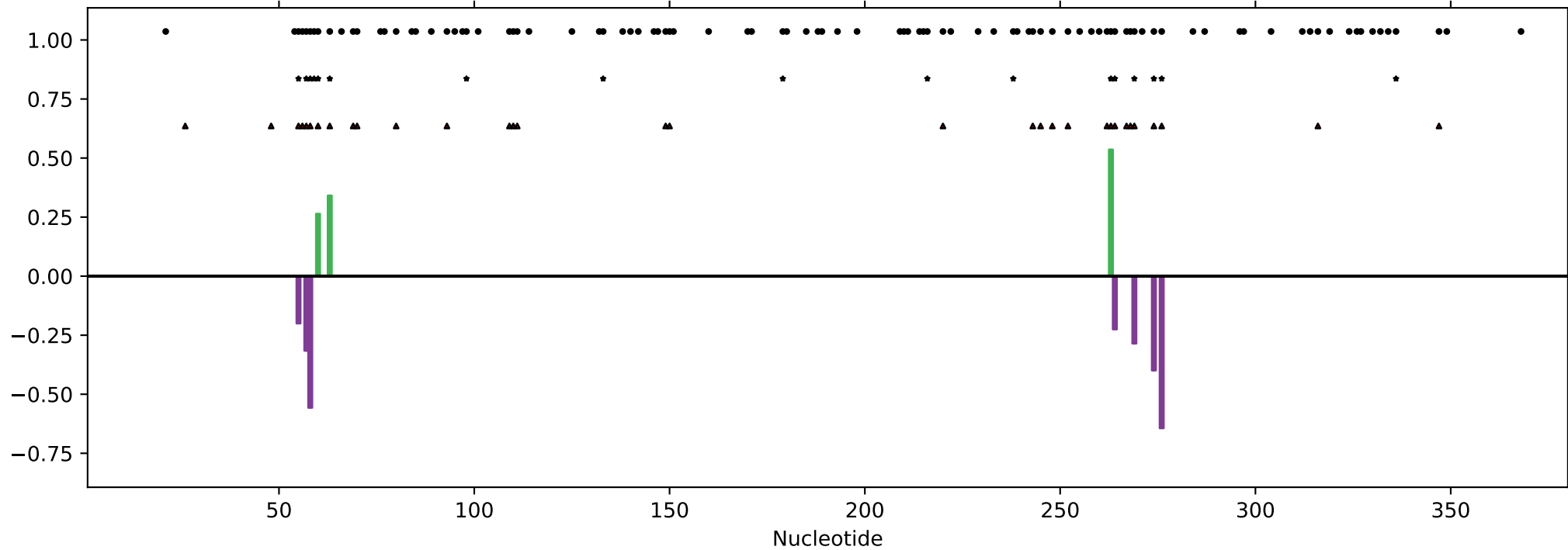

Supplement: Supplementary file 7 — Statistical deltaSHAPE analysis. [file 41594_2022_746_MOESM7_ESM.zip › deltaSHAPE/threeG_dimer_vs_oneGtwoG_monomer.pdf]

$\Delta$ SHAPE

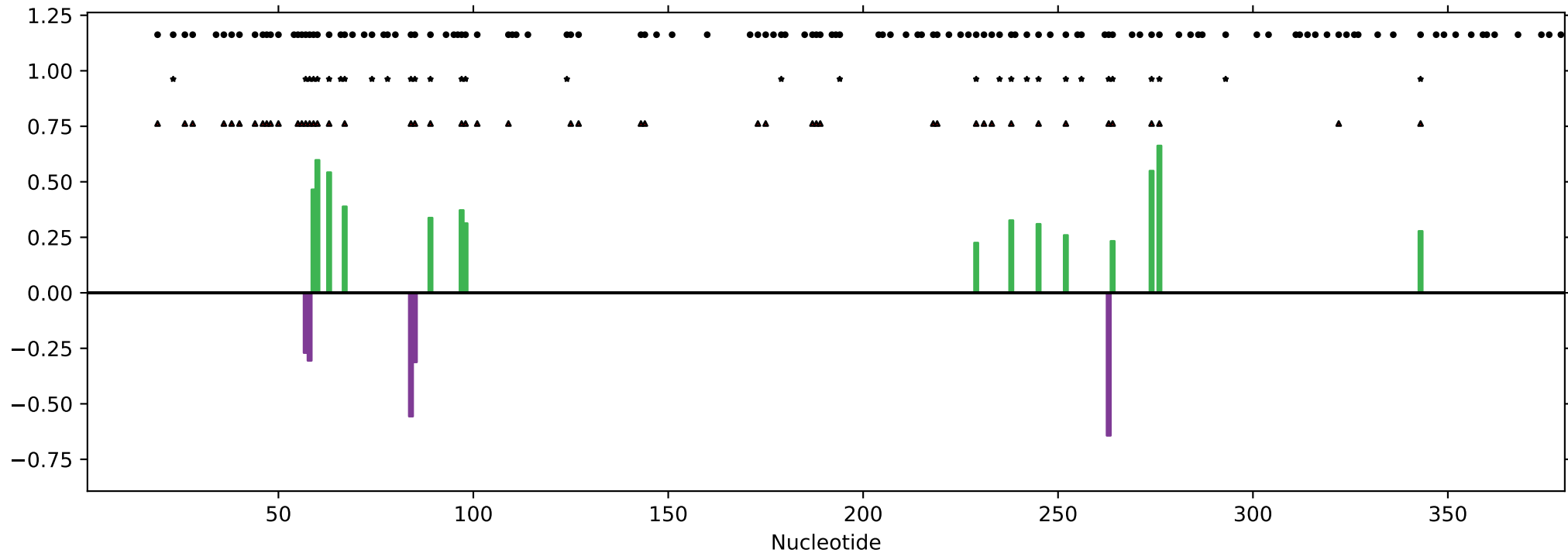

Supplement: Supplementary file 7 — Statistical deltaSHAPE analysis. [file 41594_2022_746_MOESM7_ESM.zip › deltaSHAPE/threeG_monomer_vs_oneGtwoG_dimer.pdf]

$\Delta$ SHAPE

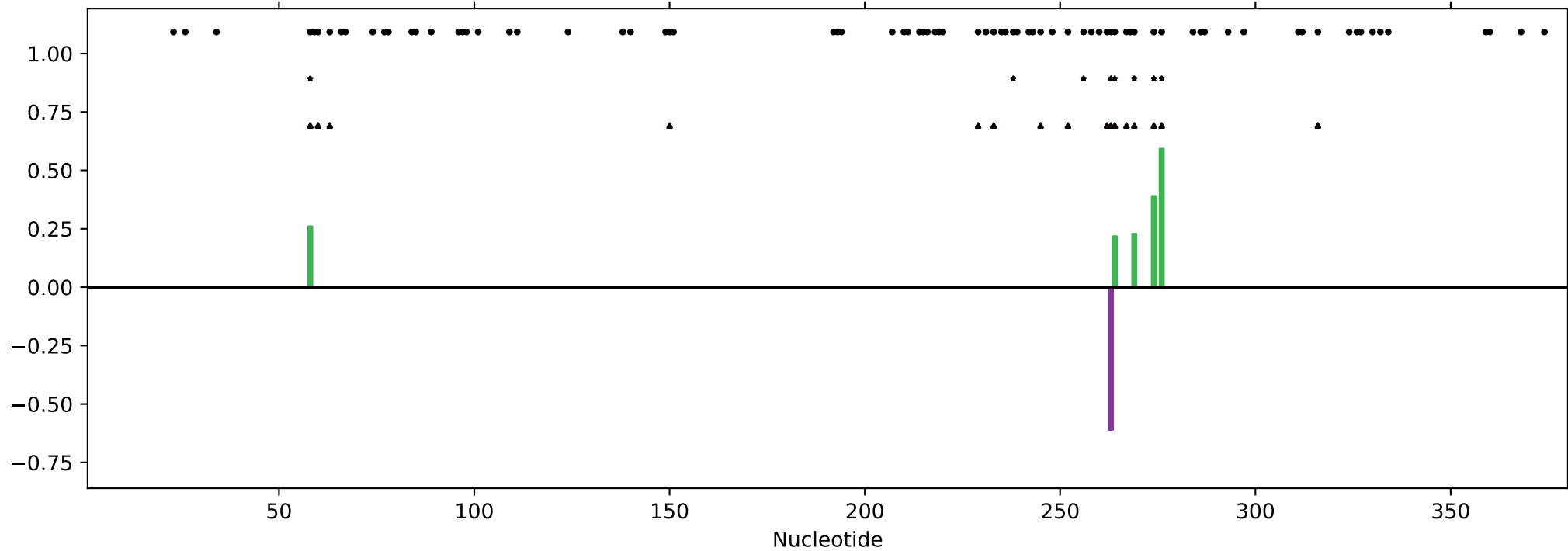

Supplement: Supplementary file 7 — Statistical deltaSHAPE analysis. [file 41594_2022_746_MOESM7_ESM.zip › deltaSHAPE/oneGtwoG_monomer_vs_oneGtwoG_dimer.pdf]

$\Delta$ SHAPE

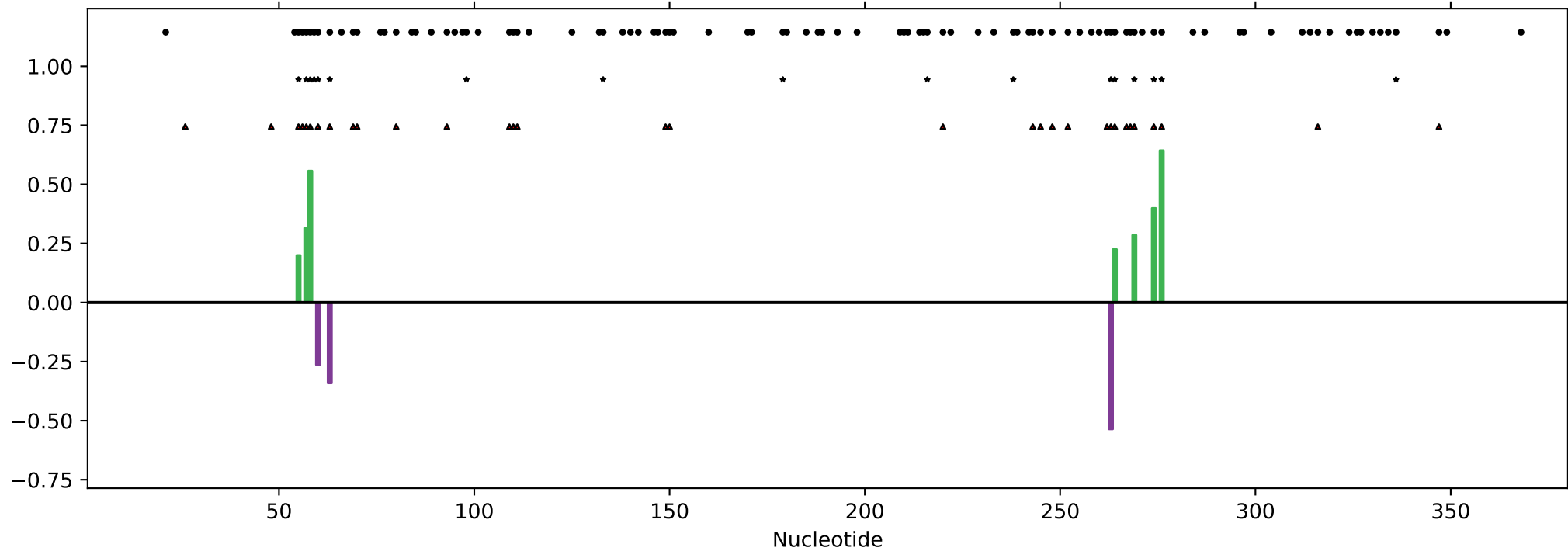

Supplement: Supplementary file 7 — Statistical deltaSHAPE analysis. [file 41594_2022_746_MOESM7_ESM.zip › deltaSHAPE/oneGtwoG_monomer_vs_threeG_dimer.pdf]

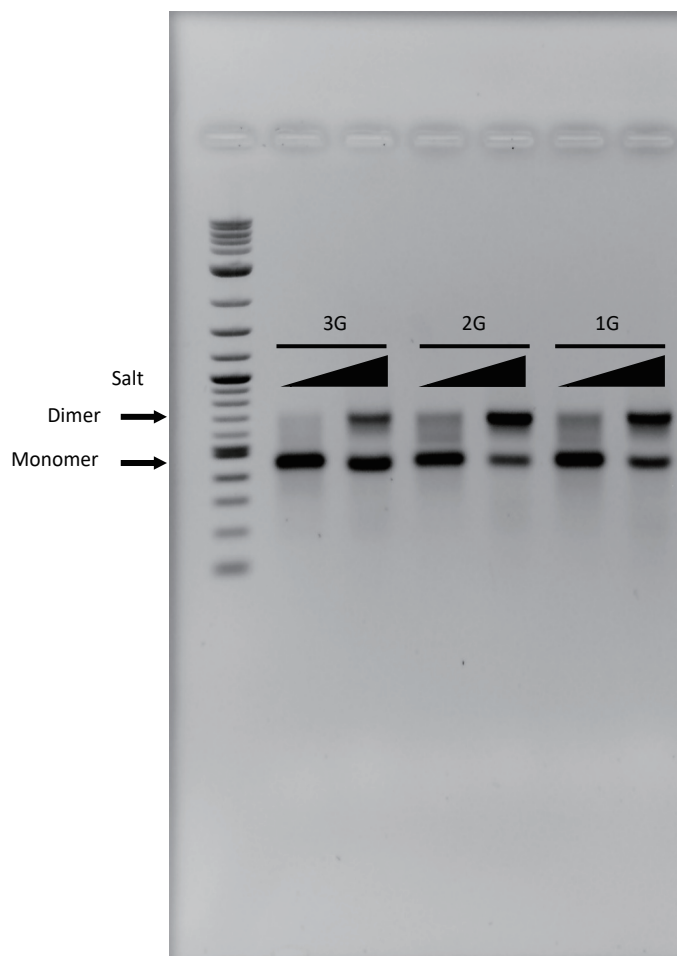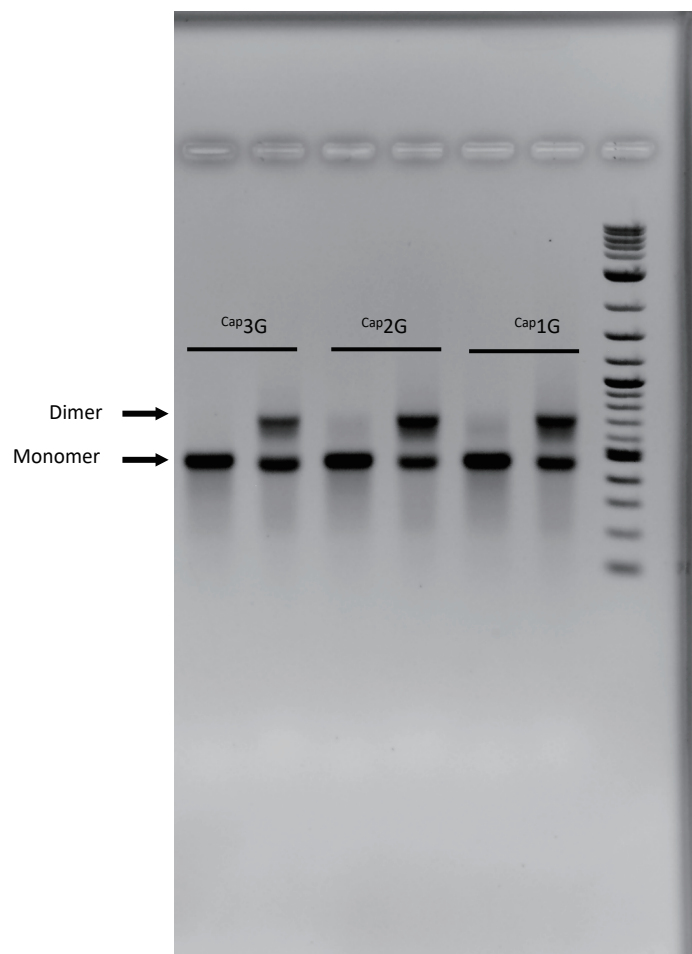

Supplement: Source Data Fig. 2 — Uncropped gels for Fig. 2a. Source data for Fig. 2b–f. [file 41594_2022_746_MOESM14_ESM.zip › Figure 2/figure2a_gels.pdf]

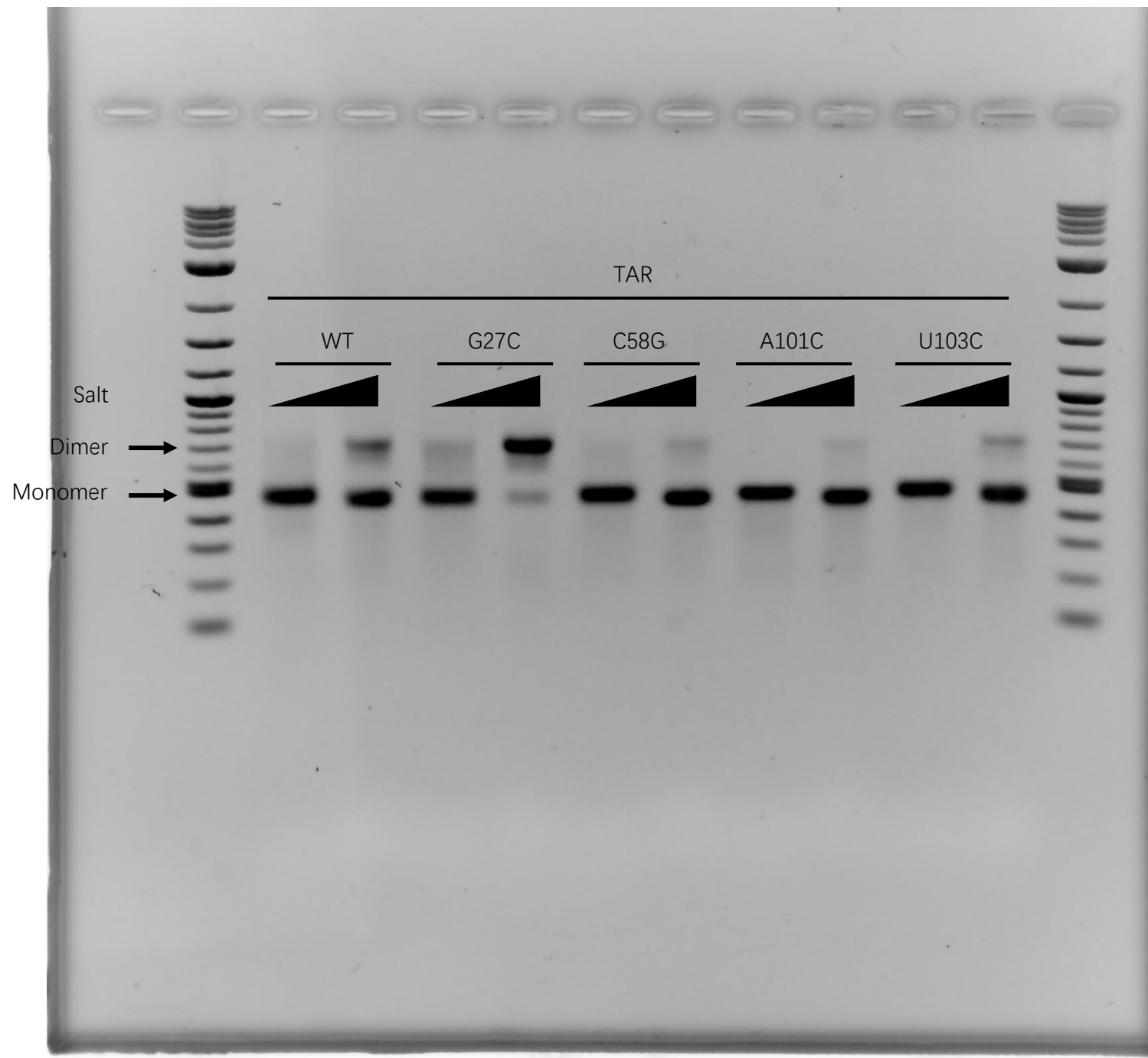

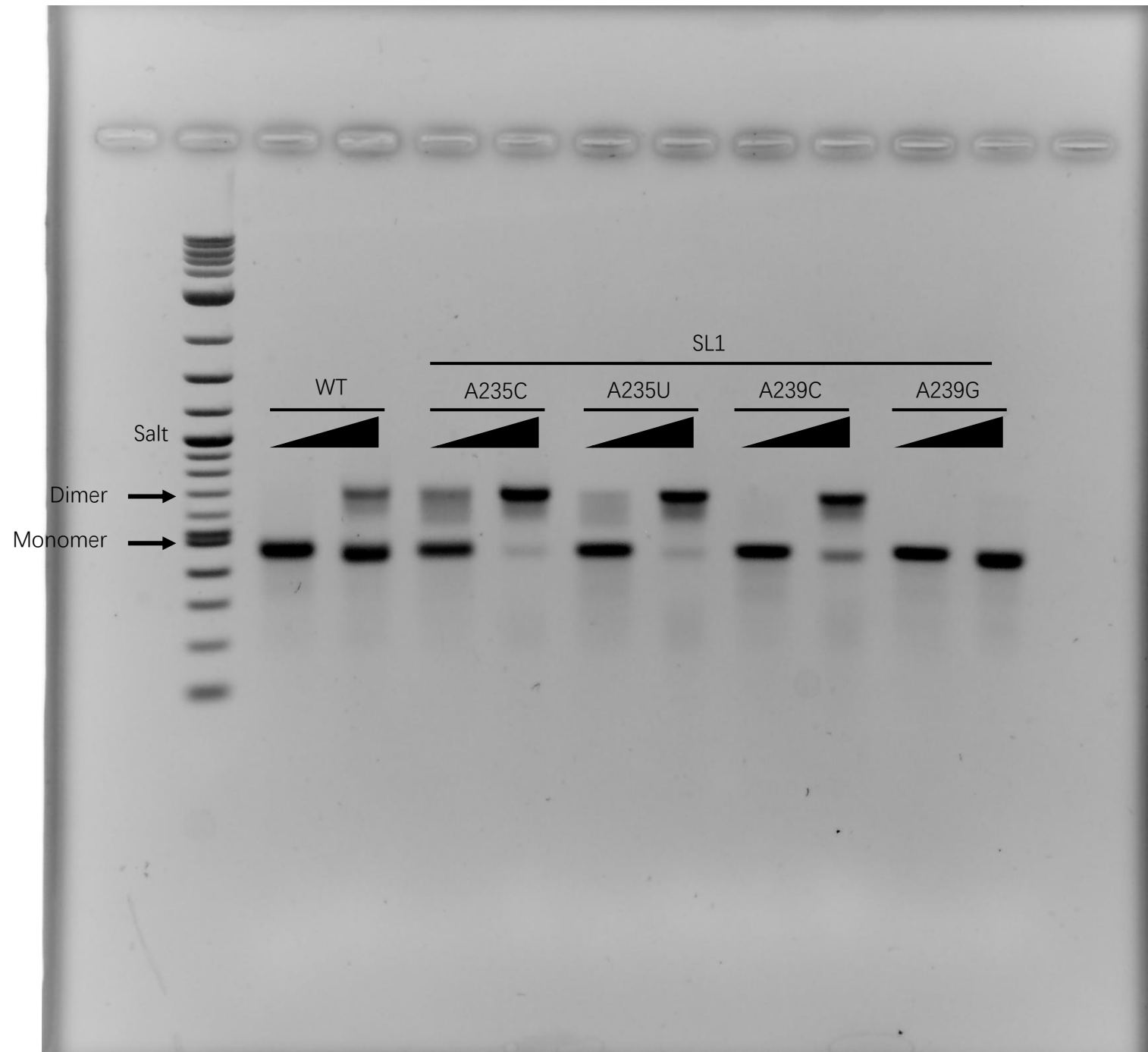

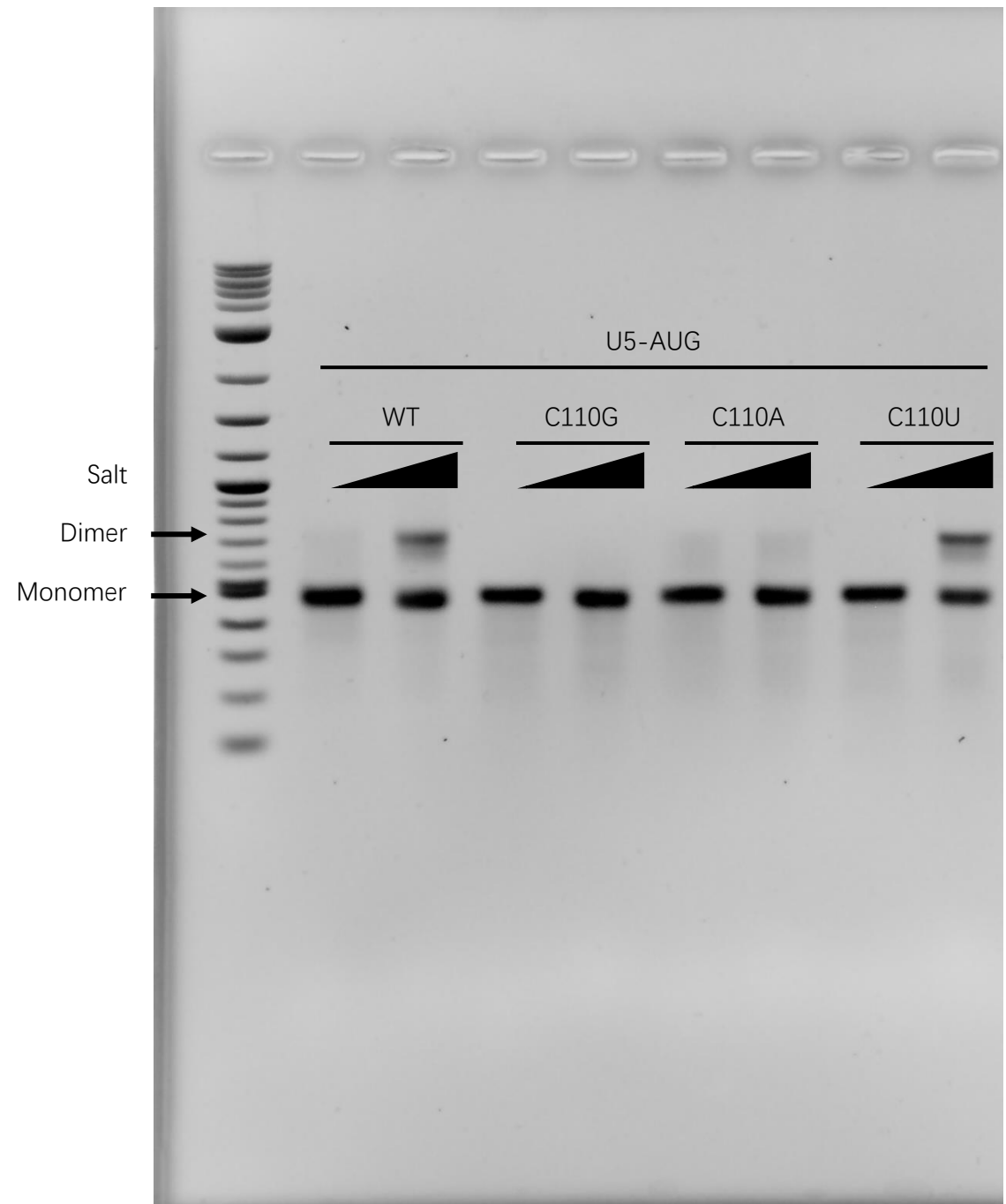

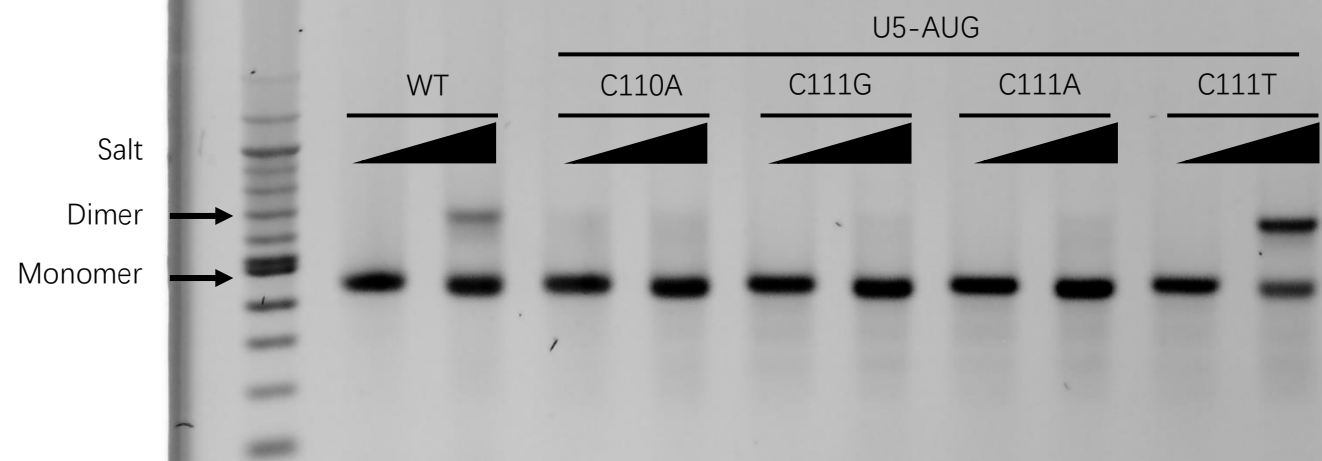

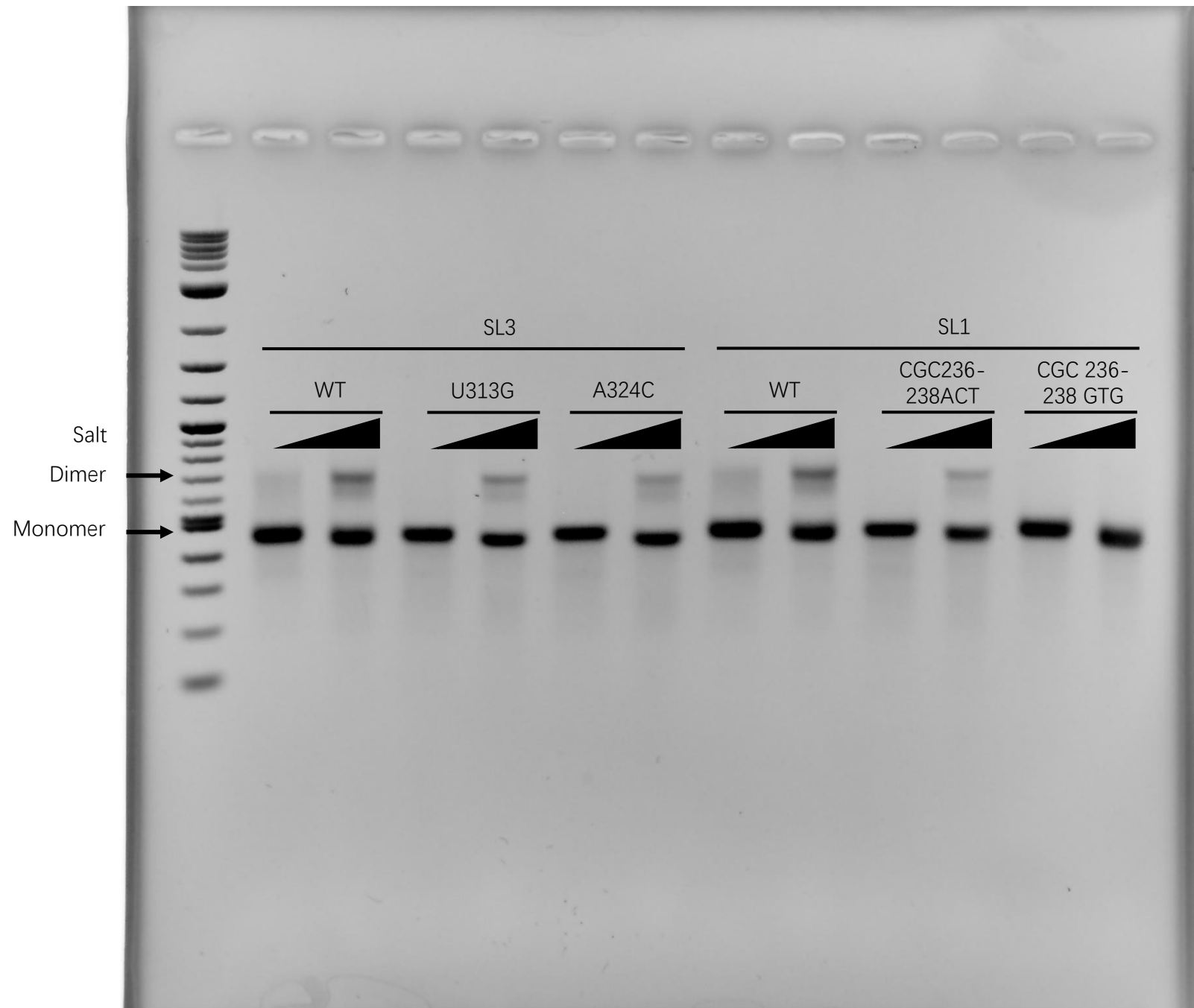

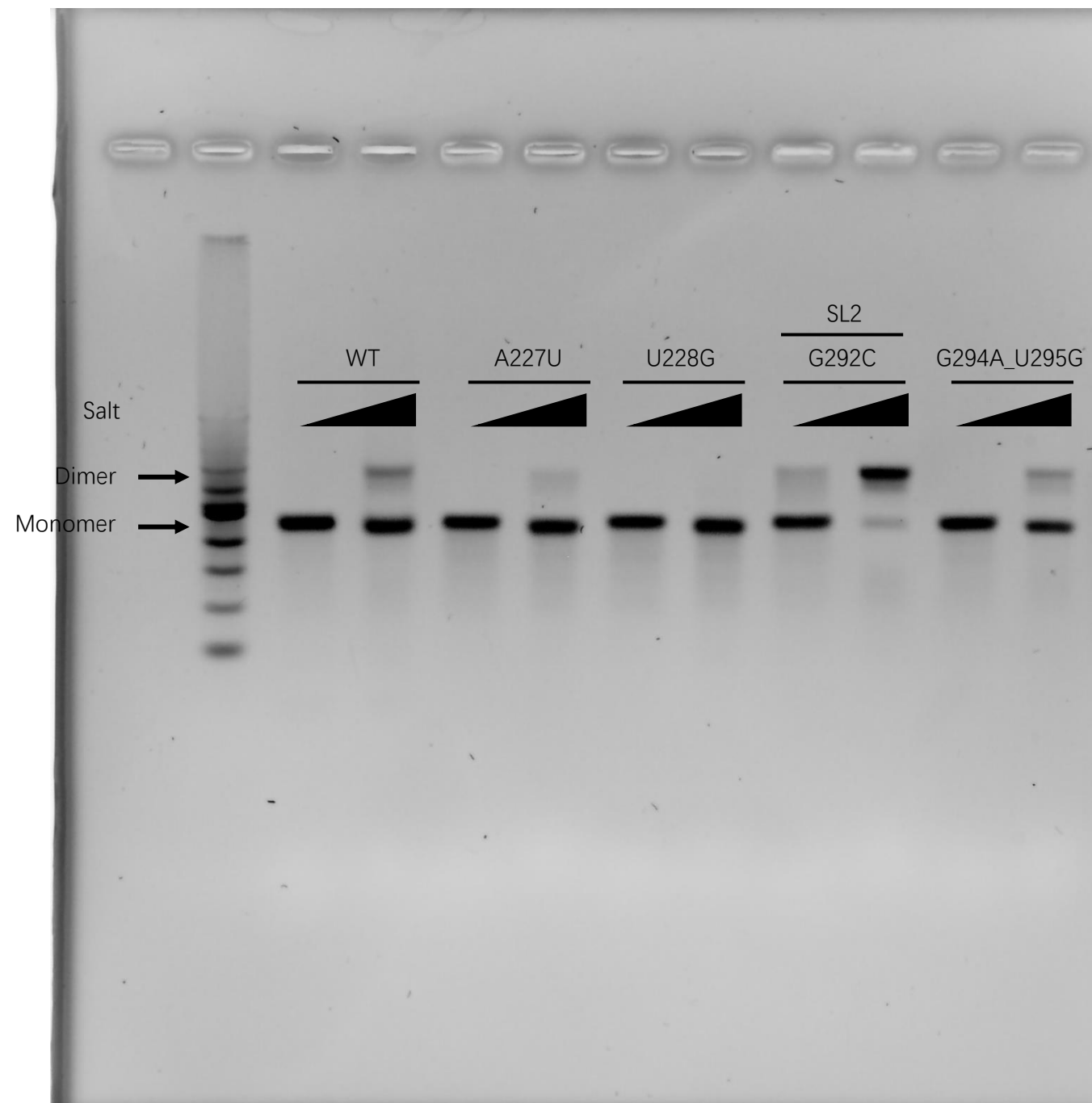

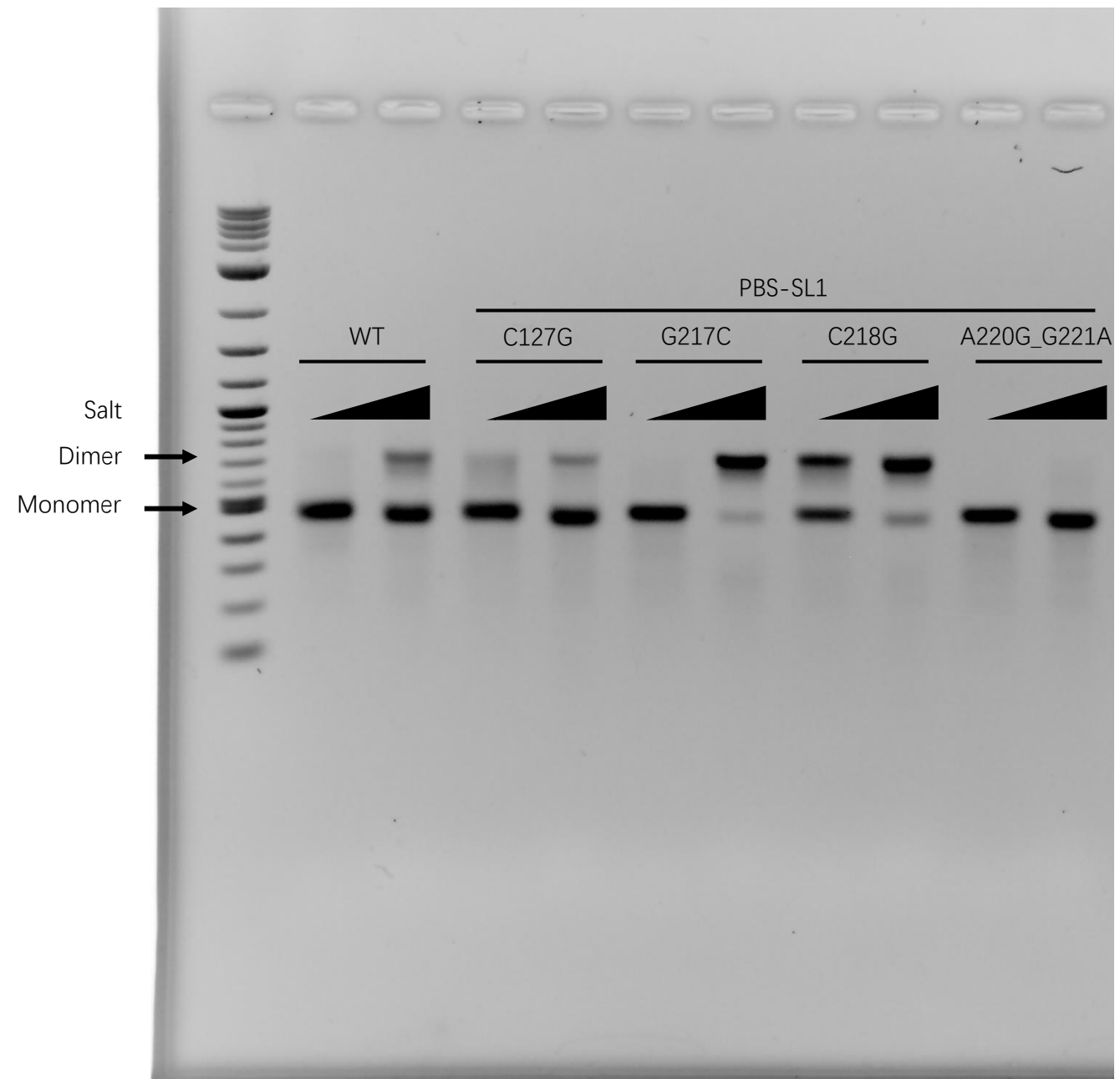

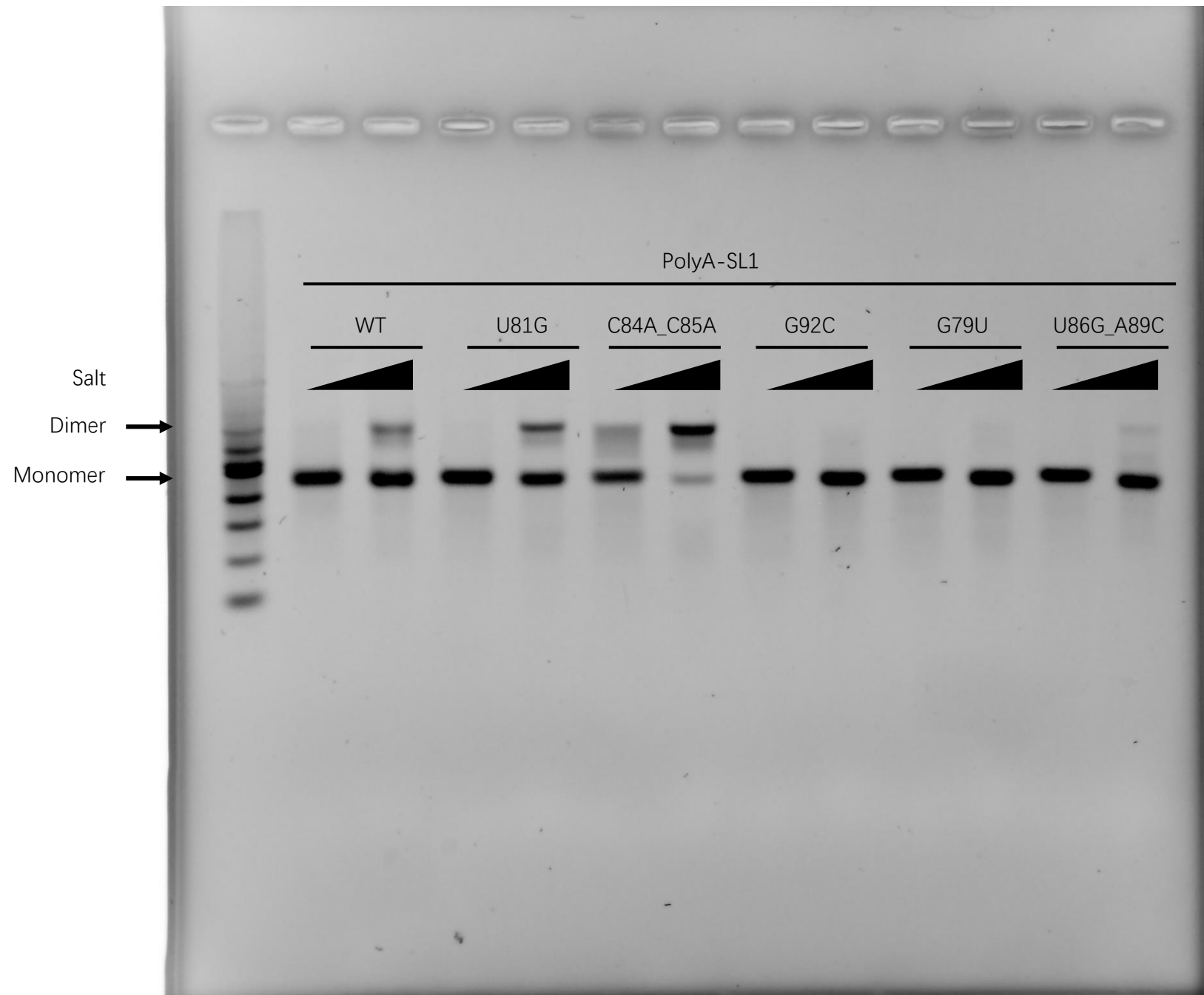

Supplement: Source Data Fig. 6 — Uncropped gels for Fig. 6a. Source data for color plotting on structure in Fig. 6. [file 41594_2022_746_MOESM18_ESM.zip › Figure 6/figure6a_gels.pdf]

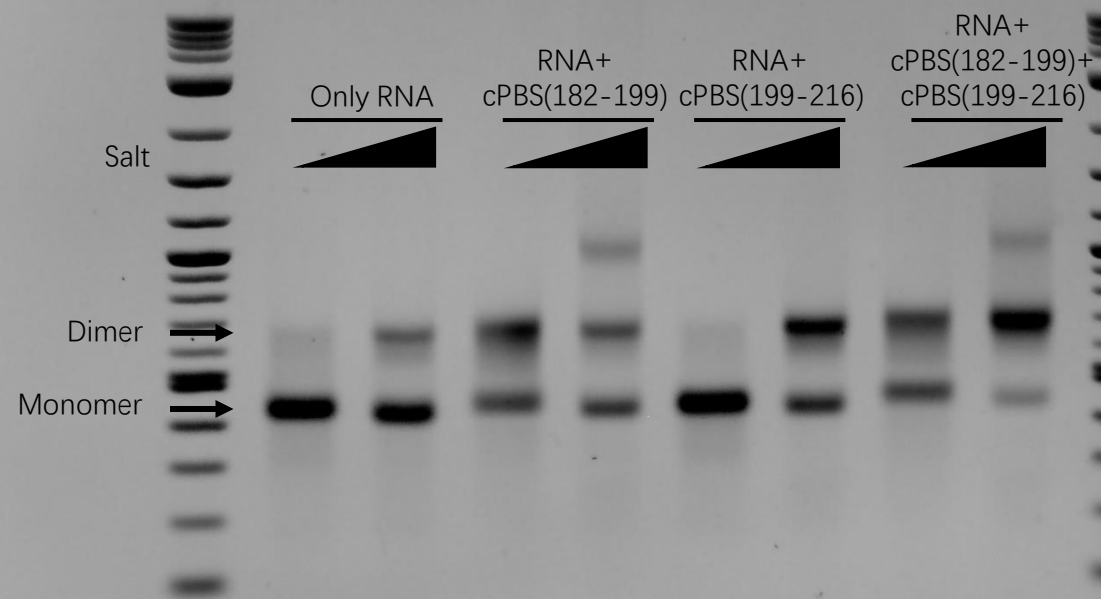

Supplement: Source Data Fig. 7 — Uncropped gels for Fig. 7a. Excel file of competition assay results. Excel file of MST analysis for Fig. 7b. [file 41594_2022_746_MOESM19_ESM.zip › Figure 7/figure7a.pdf]
