# Supplementary material for: Short- and long-range interactions in the HIV-1 5′ UTR regulate genome dimerization and packaging
Source: Nat Struct Mol Biol. 2022 Mar 28;29(4):306–19. doi: 10.1038/s41594-022-00746-2 (PMC9010304; doi:10.1038/s41594-022-00746-2)
Supplement: Supplementary file 5 — Two-dimensional structure prediction for unpooled samples. [file 41594_2022_746_MOESM5_ESM.zip › 2d-structure-inference/z_score/threeG_monomer_z_score.pdf]

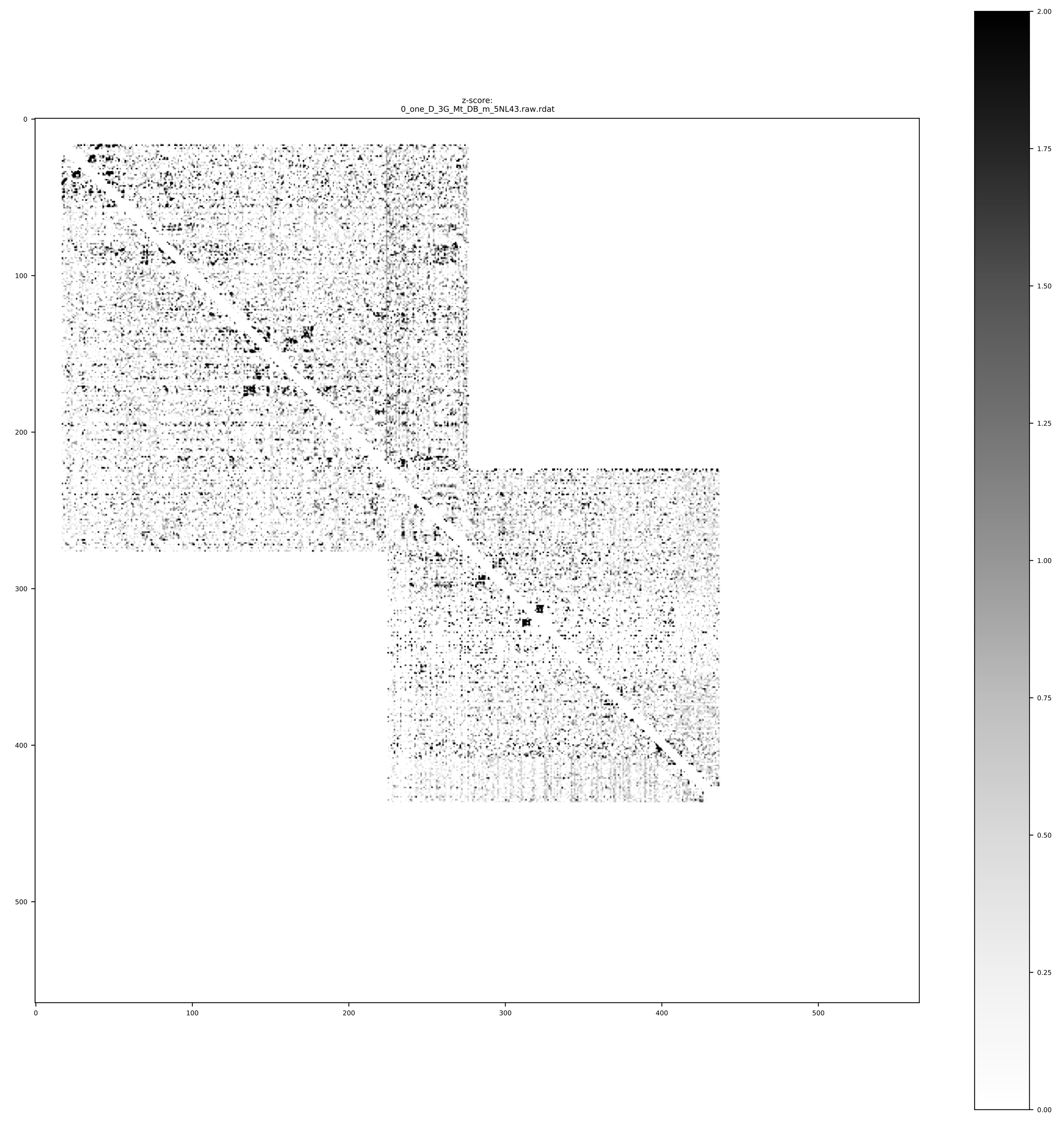
z-score:
0_one_D_3G_Mt_DB_m_5NL43.raw.rdat
0
100
200
300
400
500
0.00
0.25
0.50
0.75
1.00
1.25
1.50
1.75
2.00

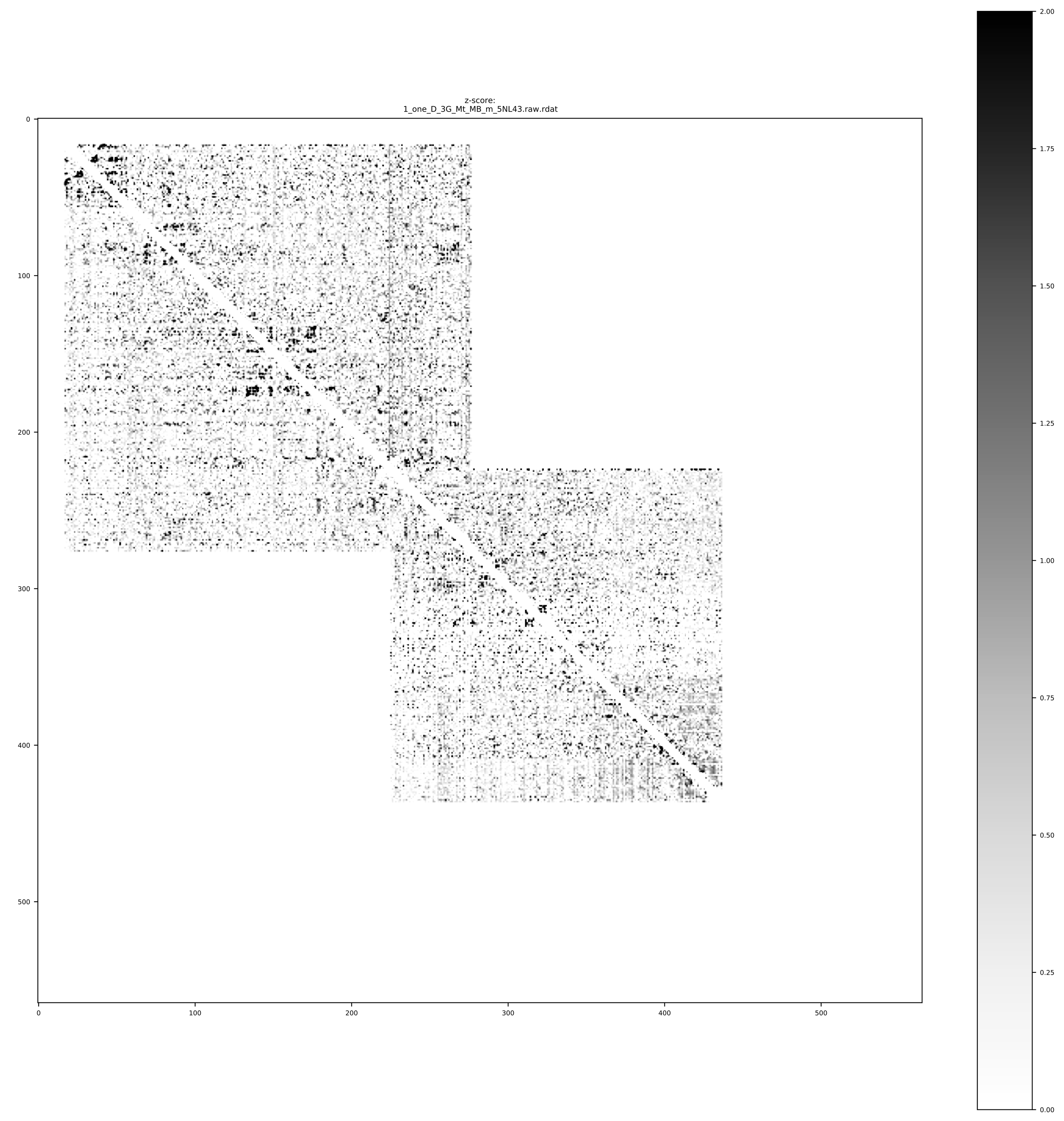
z-score:
1_one_D_3G_Mt_MB_m_5NL43.raw.rdat
0
100
200
300
400
500
0.00
0.25
0.50
0.75
1.00
1.25
1.50
1.75
2.00

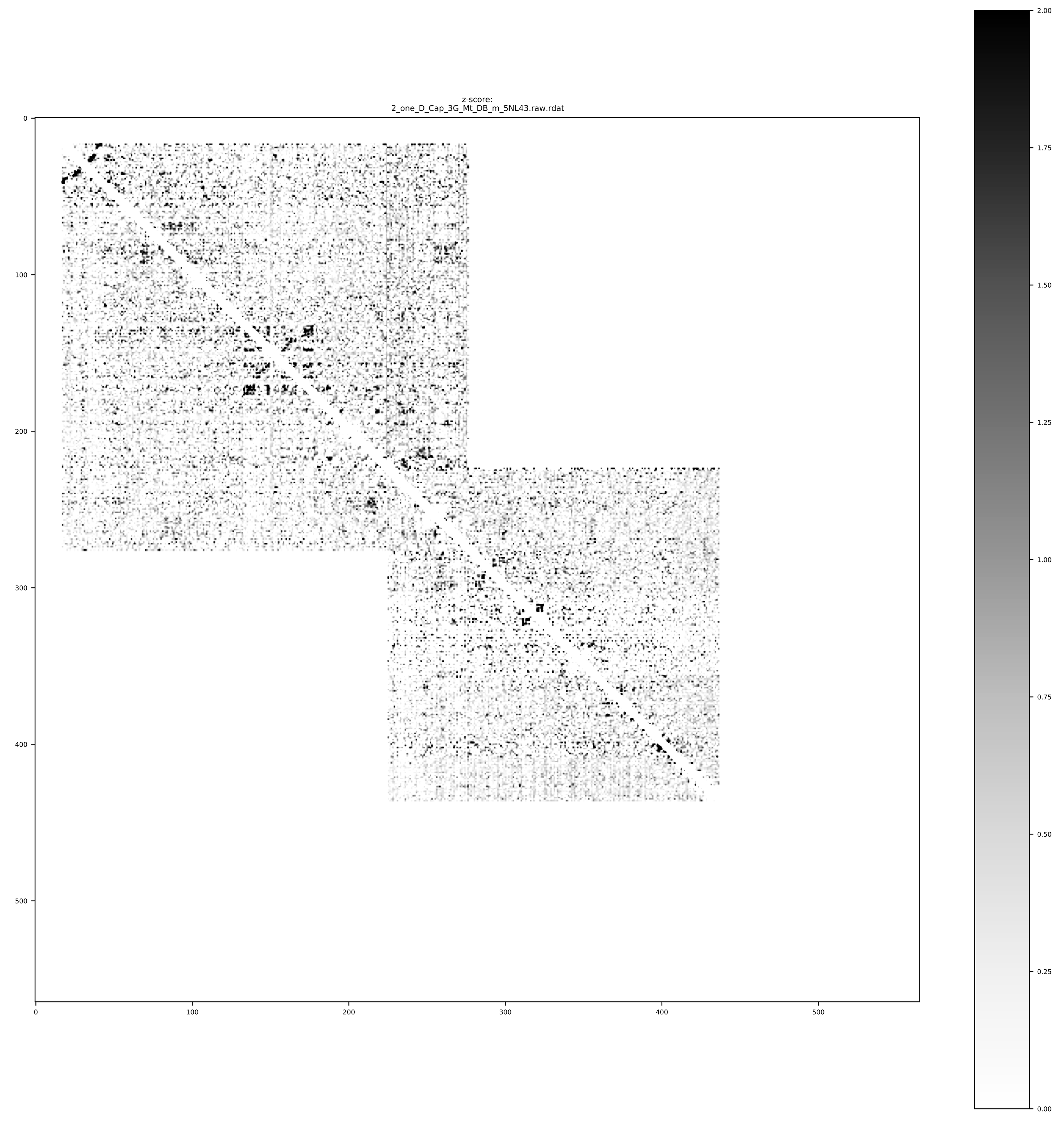
z-score:
2_one_D_Cap_3G_Mt_DB_m_5NL43.raw.rdat
0
100
200
300
400
500
0.00
0.25
0.50
0.75
1.00
1.25
1.50
1.75
2.00

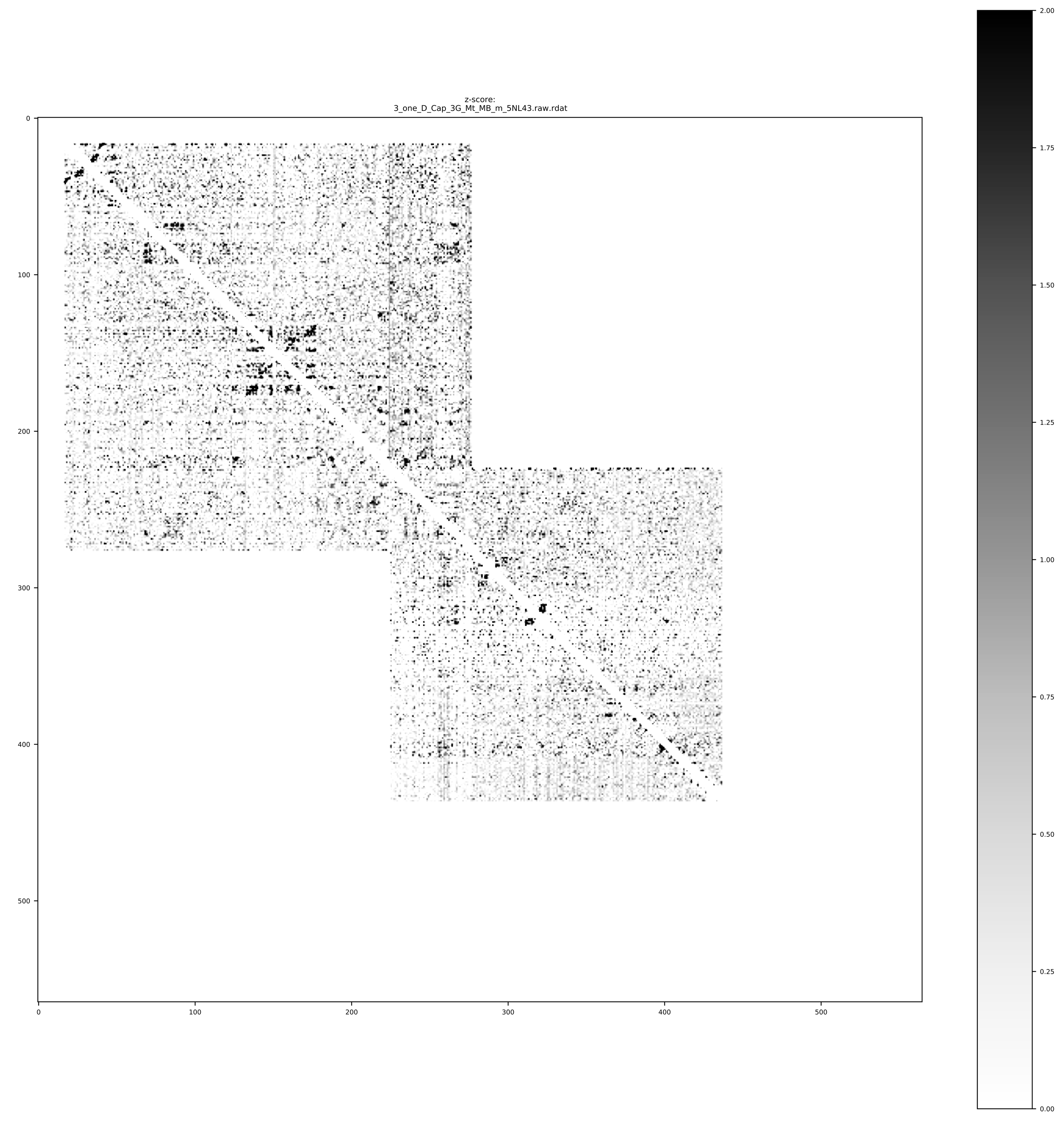
z-score:
3_one_D_Cap_3G_Mt_MB_m_5NL43.raw.rdat
0
100
200
300
400
500
0.00
0.25
0.50
0.75
1.00
1.25
1.50
1.75
2.00
