# Supplementary material for: Short- and long-range interactions in the HIV-1 5′ UTR regulate genome dimerization and packaging
Source: Nat Struct Mol Biol. 2022 Mar 28;29(4):306–19. doi: 10.1038/s41594-022-00746-2 (PMC9010304; doi:10.1038/s41594-022-00746-2)
Supplement: Supplementary file 5 — Two-dimensional structure prediction for unpooled samples. [file 41594_2022_746_MOESM5_ESM.zip › 2d-structure-inference/best_helix/one_two_G_dimer_best_helix.pdf]

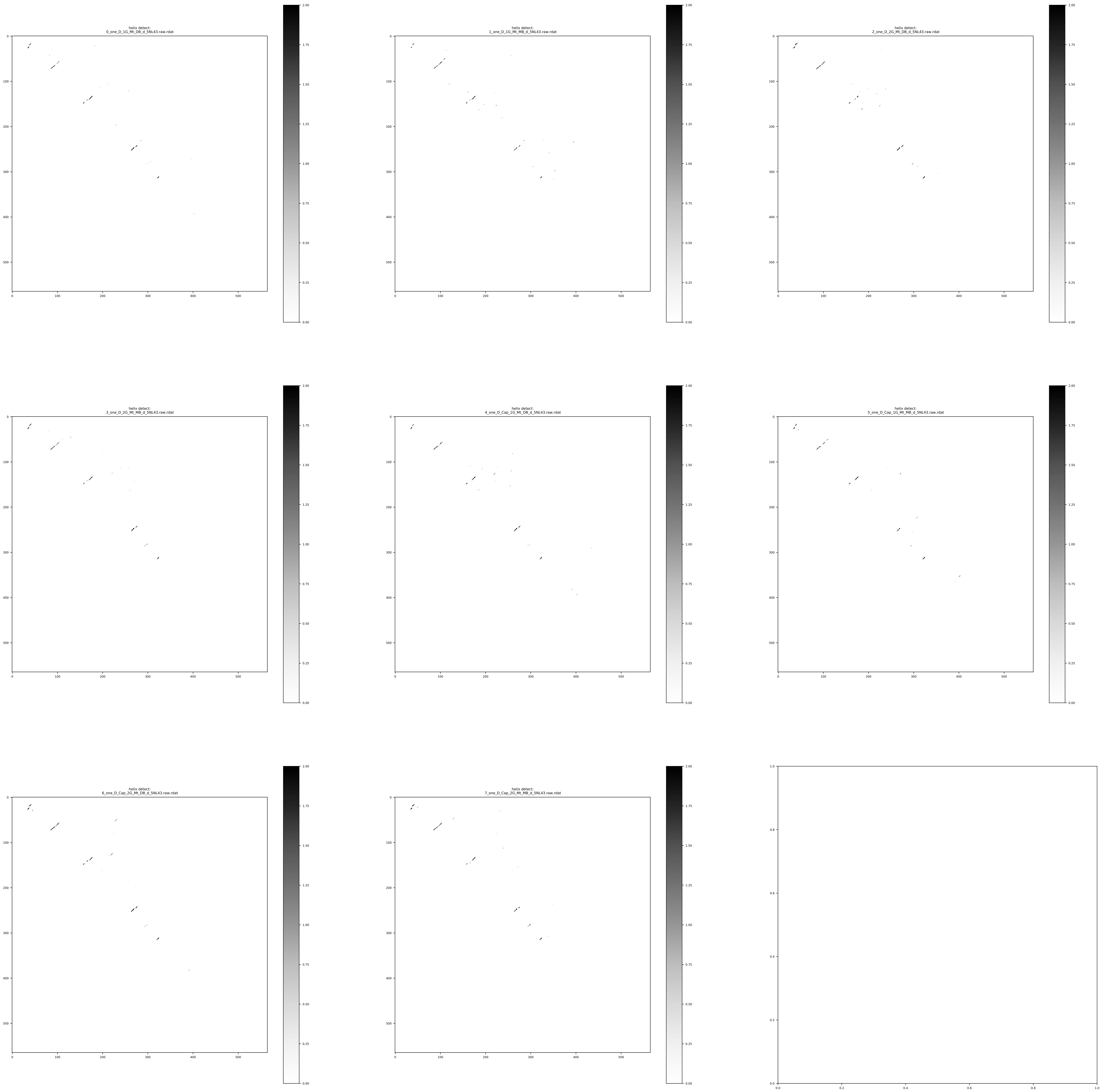
helix detect:
0_one_D_1G_Mt_DB_d_5NL43.raw.rdat
helix detect:
1_one_D_1G_Mt_MB_d_5NL43.raw.rdat
helix detect:
2_one_D_2G_Mt_DB_d_5NL43.raw.rdat
helix detect:
3_one_D_2G_Mt_MB_d_5NL43.raw.rdat
helix detect:
4_one_D_Cap_1G_Mt_DB_d_5NL43.raw.rdat
helix detect:
5_one_D_Cap_1G_Mt_MB_d_5NL43.raw.rdat
helix detect:
6_one_D_Cap_2G_Mt_DB_d_5NL43.raw.rdat
helix detect:
7_one_D_Cap_2G_Mt_MB_d_5NL43.raw.rdat
