# Supplementary material for: Short- and long-range interactions in the HIV-1 5′ UTR regulate genome dimerization and packaging
Source: Nat Struct Mol Biol. 2022 Mar 28;29(4):306–19. doi: 10.1038/s41594-022-00746-2 (PMC9010304; doi:10.1038/s41594-022-00746-2)
Supplement: Supplementary file 5 — Two-dimensional structure prediction for unpooled samples. [file 41594_2022_746_MOESM5_ESM.zip › 2d-structure-inference/convolution_filtered/threeG_monomer_conv_filter.pdf]

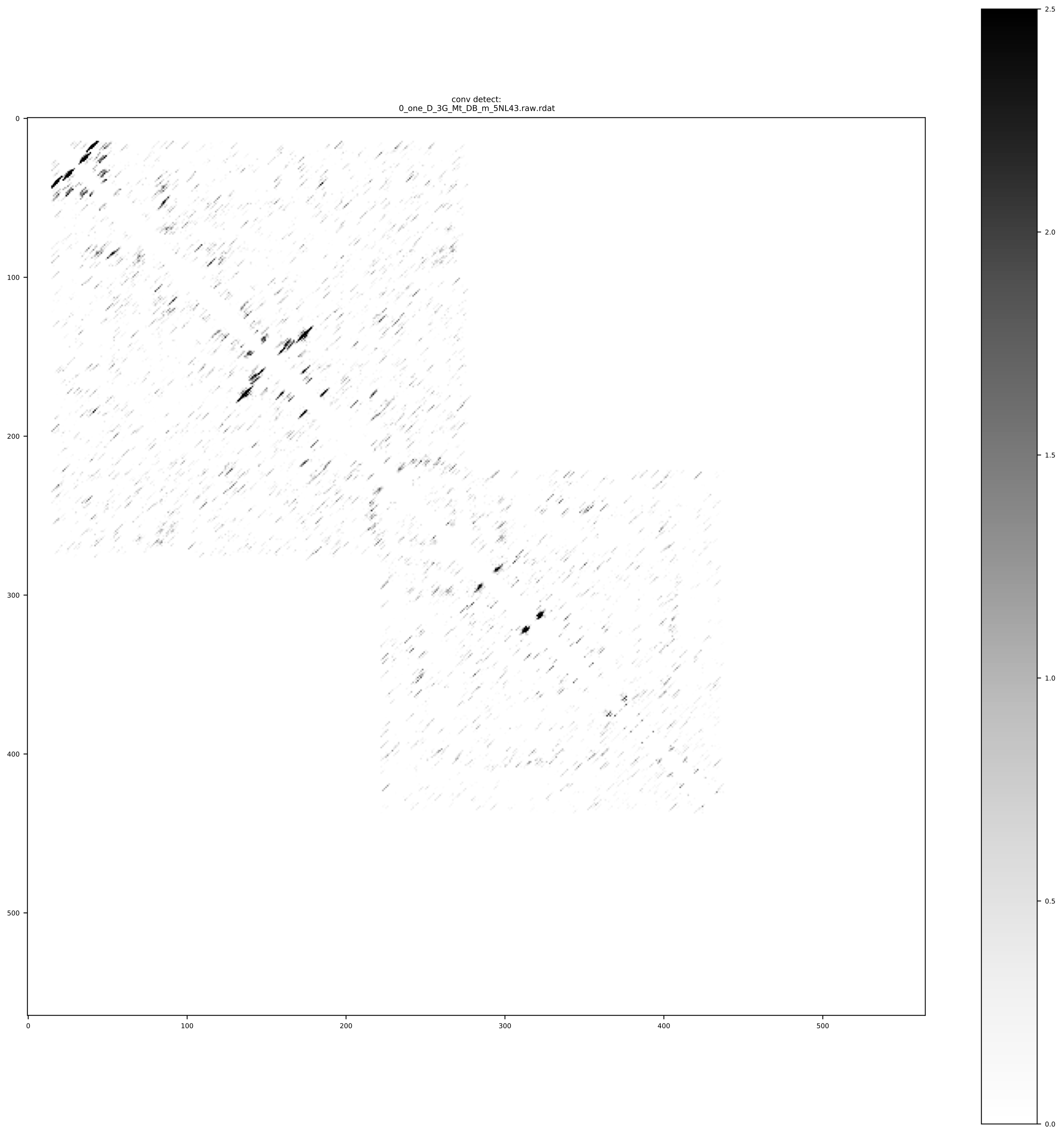
conv detect:
0_one_D_3G_Mt_DB_m_5NL43.raw.rdat
0
100
200
300
400
500
0.0
0.5
1.0
1.5
2.0
2.5

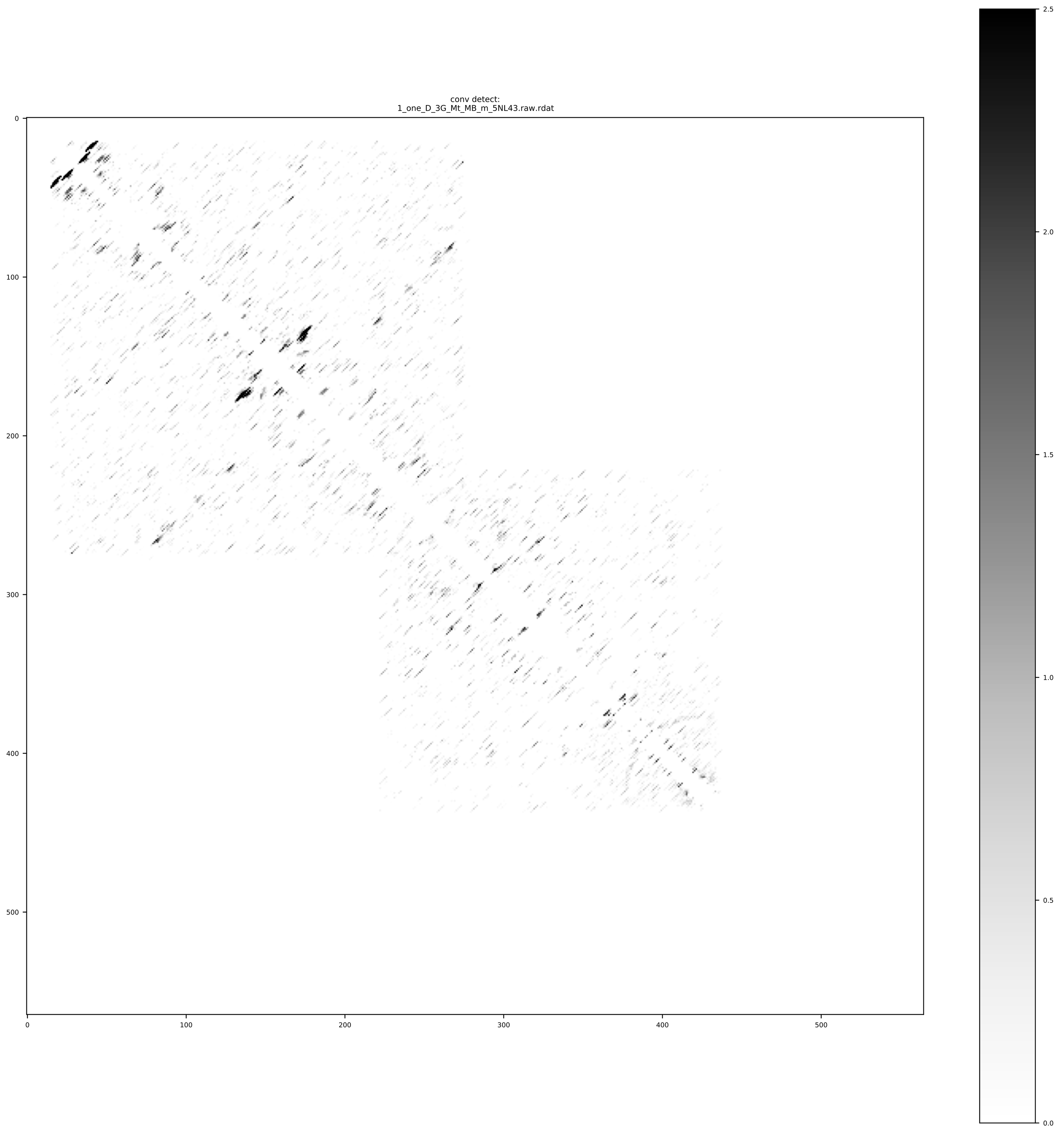
conv detect:
1_one_D_3G_Mt_MB_m_5NL43.raw.rdat
0
100
200
300
400
500
0.0
0.5
1.0
1.5
2.0
2.5

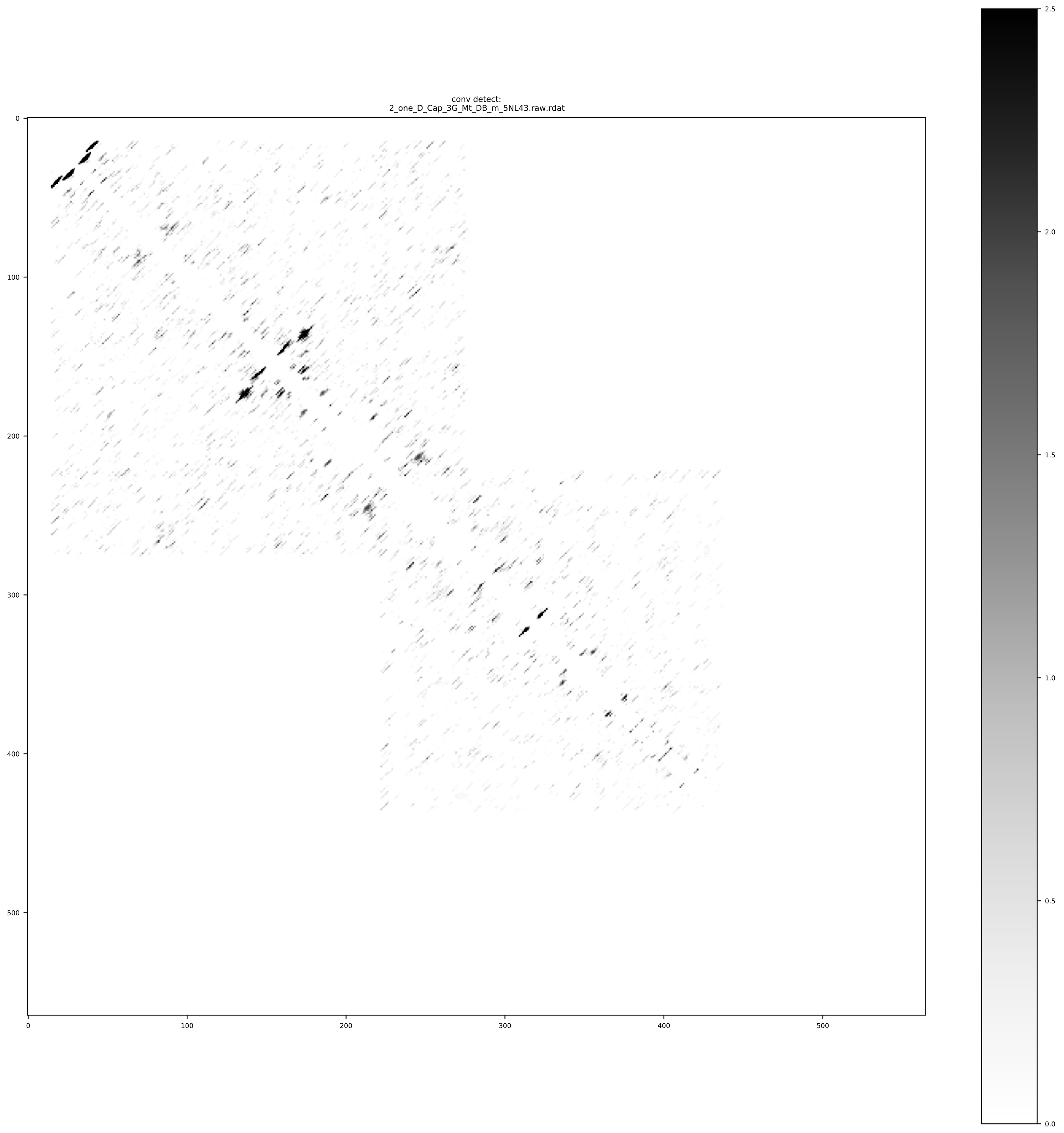
conv detect:
2_one_D_Cap_3G_Mt_DB_m_5NL43.raw.rdat
0
100
200
300
400
500
0.0
0.5
1.0
1.5
2.0
2.5

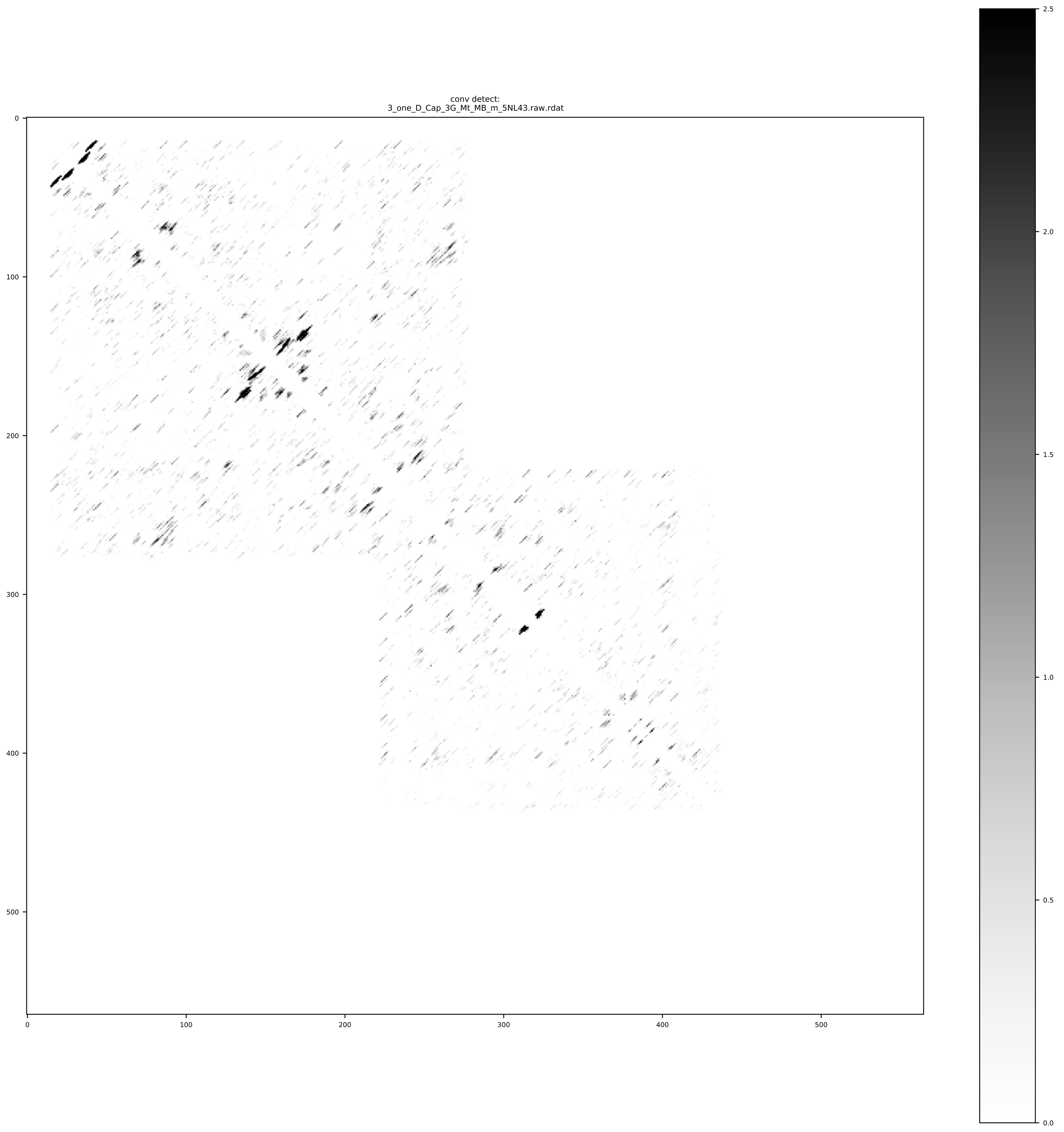
conv detect:
3_one_D_Cap_3G_Mt_MB_m_5NL43.raw.rdat
0
100
200
300
400
500
0.0
0.5
1.0
1.5
2.0
2.5
